# High Entropy Alloy Thin Films as Efficient Spin‐Orbit Torque Sources for Spintronic Memories

**DOI:** 10.1002/adma.202416820

**Published:** 2025-06-04

**Authors:** Peng Wang, Andrea Migliorini, Yung‐Cheng Li, Hakan Deniz, Ilya Kostanovski, Jae‐Chun Jeon, Stuart S. P. Parkin

**Affiliations:** ^1^ Max Planck Institute of Microstructure Physics 06120 Halle (Saale) Germany; ^2^ Institute of Physics Martin Luther University Halle‐Wittenberg 06120 Halle (Saale) Germany

**Keywords:** high entropy alloy, racetrack memory, spin Hall effect, spintronics

## Abstract

High entropy alloys (HEAs) containing multiple elements are emerging as advanced materials with enhanced functionalities. However, their use for spintronic applications remains elusive. Here, it is demonstrated that iridium based HEAs, grown by magnetron sputtering at room temperature, can be used as spin Hall layers. These films display highly efficient conversion of charge current into spin current. They also allow for the epitaxial growth of magnetic multilayers with perpendicular magnetic anisotropy as well as synthetic antiferromagnets using a ternary RuAlGa antiferromagnetic coupling layer. It is demonstrated that iridium‐based HEAs serve as effective sources of spin‐orbit torque, as quantified by spin‐torque ferromagnetic resonance and harmonic Hall measurements, enabling current‐induced magnetization reversal and domain wall motion. The threshold current density for current‐induced magnetization switching is found to be as low as ∼10 MA cm^−2^ with reproducible deterministic switching, and that domain walls in HEA‐based racetracks can be driven at speeds of up to 300 m s^−1^ at a current density of 65 MA cm^−2^. These results show that HEAs should be considered for high‐performance spintronic applications.

## Graphical abstract

High‐entropy alloys with significantly diluted heavy metal content are found to be a very efficient source of spin‐orbit torques for spintronic applications. Epitaxially‐grown 4‐, 5‐, 6‐element alloy thin films are successfully integrated in magnetic racetrack memory devices, showing efficient domain wall motion, thereby adding a large family of materials to those that can be exploited for spin current generation.

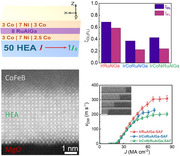

## 1 Introduction

High entropy alloys (HEAs) are atomically disordered alloys containing multiple elements, typically numbering from four to six, or even more.^[^ ^1^, ^2^ ^]^ Such alloys have been shown to have superior properties, including mechanical strength,^[^ ^3^, ^4^, ^5^ ^]^ thermal stability,^[^ ^6^, ^7^ ^]^ and biocompatibility,^[^ ^8^, ^9^ ^]^ and are, thus, good candidates for challenging applications. Many of their physical properties have been studied in detail^[^ ^4^, ^5^, ^6^, ^10^, ^11^, ^12^, ^13^ ^]^ but there have been very few reports concerning their applications in spintronics.^[^ ^14^ ^]^ In particular, spintronic non‐volatile memories are composed of complex thin film multilayered structures. These structures include magnetic layers that form the storage elements. Even more important are thin layers that convert charge currents to spin currents that are used to set the magnetic state of the memory.^[^ ^15^, ^16^, ^17^, ^18^ ^]^ The conversion efficiency of charge into spin current is characterized by the spin Hall angle (θ_SH_).^[^ ^15^ ^]^ The generated spin current injected into an adjacent magnetic thin film gives rise to spin‐orbit torques (SOTs),^[^ ^19^, ^20^, ^21^ ^]^ which have been exploited to control the magnetization direction of magnetic nano‐elements for conventional MRAM (magnetic random‐access memory),^[^ ^22^, ^23^, ^24^, ^25^ ^]^ as well as to move domain walls in magnetic racetrack devices,^[^ ^26^, ^27^ ^]^ which have the potential to enable memory and logic spintronic applications with advanced and unconventional functionalities.^[^ ^28^, ^29^, ^30^, ^31^ ^]^

Typically, the spin currents are created via a spin Hall effect (SHE) derived from some form of spin‐orbit coupling. Thus, these layers often contain heavy metals. To date the largest SHEs have been found in simple elements such as Ta,^[^ ^15^ ^]^ W,^[^ ^19^ ^]^ and Pt,^[^ ^20^ ^]^ and in binary crystalline alloys such as Ir‐Al,^[^ ^21^ ^]^ but there is only one report on the use of HEA layers.^[^ ^14^ ^]^ In particular, the implementation of HEAs for spintronic applications is of great interest thanks to their enhanced degree of freedom and nearly infinite possible combinations and compositions, which can enable a paradigm change for spintronic materials. Indeed, spintronic applications rely heavily on rare‐earth elements and heavy metals, which are needed, for example, for large SOTs. Thus, the synthesis of HEAs, in which these critical materials can be diluted with lighter and more accessible elements, could be beneficial not only for their performance, but also in terms of ease of access, economic costs, and environmental sustainability.^[^ ^2^ ^]^ Here we demonstrate this concept by reporting HEAs with a highly textured crystal structure, which are grown by magnetron sputtering at room temperature, in which Ir is increasingly diluted in 4‐, 5‐, and 6‐element alloys. We show that these HEAs can give rise to significant SOTs that can drive domain walls efficiently, thereby adding a very large family of materials to those that can be exploited for SOT‐based applications.

## 2 Results and Discussion

Herein, we use a versatile magnetron sputtering based ultra‐high vacuum deposition system to grow a series of iridium‐based HEA thin films on MgO(001) substrates at ambient temperature. Alloys with between 4 and 6 elements were explored, wherein each element is sputtered from an independent source. Ir based alloys were chosen because of previous studies that have shown large spin Hall angles in Ir‐Al binary alloys that exhibit an *L*1_0_ structure.^[^ ^21^ ^]^ We progressively dilute the heavy metal Ir by alloying it with cheaper and lighter elements, such as Co, Ni, Ru, Al and Ga. The composition of the HEA films was determined by non‐destructive Rutherford back‐scattering spectroscopy (RBS), and was tuned by changing the sputtering powers of the respective sputtering targets (see methods).

**Figure 1:**
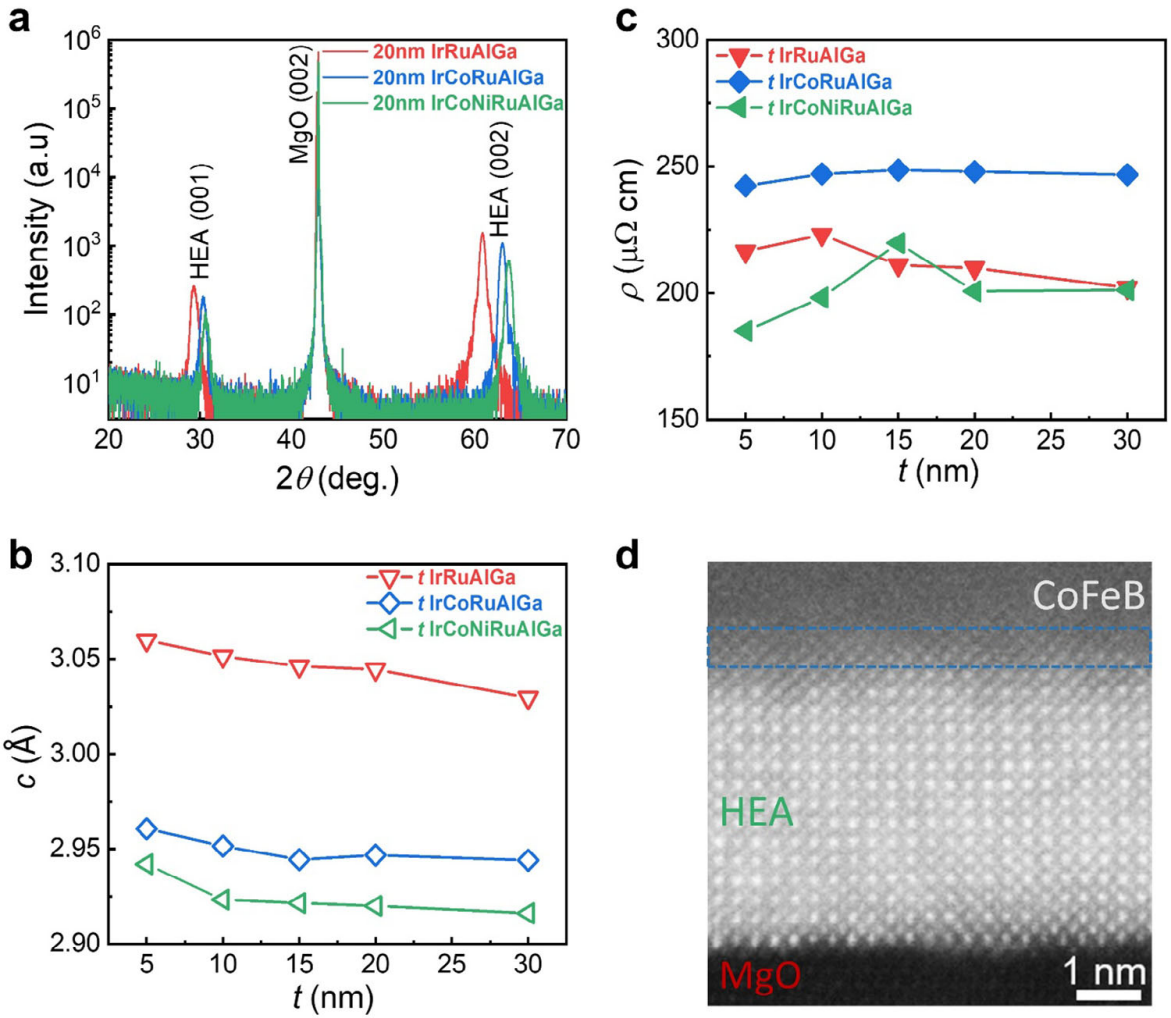
Crystal structure and electrical resistivity versus thickness for several iridium based HEA thin films. a) XRD measurements of 20 nm‐thick HEA thin films, 2θ – *ω* specular scan. b) Out‐of‐plane lattice parameter and c) electrical resistivity versus thicknesses of HEA thin films with three compositions shown in the plot. d) Cross‐sectional, high‐angle annular dark‐field transmission electron microscopy (HAADF‐STEM) image of a 5 nm thick HEA layer (IrCoNiRuAlGa) grown on MgO(001). A Co_20_Fe_60_B_20_ layer is deposited on top of the HEA Iayer. The boundary between the HEA layer and the Co_20_Fe_60_B_20_ layer is indicated by the blue dashed lines.

**Figure** **1**a shows 2θ–*ω* specular X‐ray diffraction (XRD) measurements for 20‐nm‐thick HEA films. The crystal structure of the films displays an alternating layered tetragonal structure, as can be deduced from the (00L) (L = 1, 2, 3….) XRD peaks. The absence of additional XRD peaks indicates that the film is highly crystalline and in a single‐phase HEA state. XRD measurements as a function of thickness of a typical set of films are shown in Figure S4 (Supporting Information). As shown in Figure 1b, the out‐of‐plane lattice parameter (*c*) gradually decreases with increasing film thickness indicating the relaxation of strain imposed by epitaxy with the substrate. The electrical resistivity of the films varies only slightly with thickness, as shown in Figure 1c. A representative high‐resolution transmission electron microscopy (HR‐TEM) image of the 6‐element HEA composed of IrCoNiRuAlGa is shown in Figure 1d. The image shows the high quality crystalline structure of the HEA layer and its epitaxial growth on the MgO(001) substrate. Energy dispersive X‐ray (EDX) spectroscopy elemental mapping from the HR‐TEM studies for three 5‐nm‐thick HEA films confirms the uniformity of the sputtered HEA layers (Figures S1–S3, Supporting Information).

**Figure 2:**
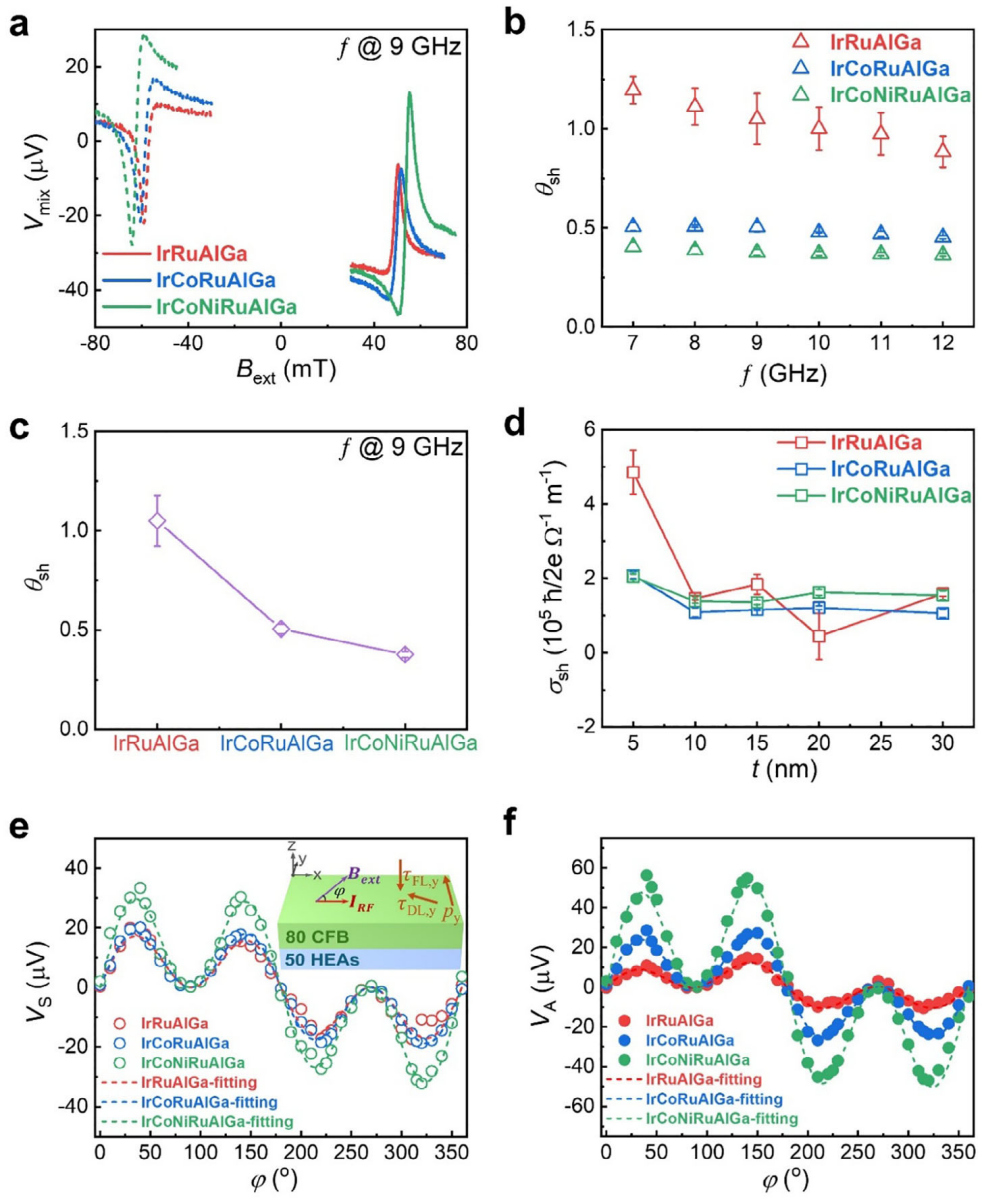
Spin‐torque ferromagnetic resonance measurements in bilayer thin films of 5 nm HEA | 8 nm Co_20_Fe_60_B_20_. a) ST‐FMR spectra measured at *f =* 9 GHz for several HEAs. b) Effective spin Hall angle as a function of *f* from 7 to 12 GHz. c) Effective spin Hall angle obtained at 9 GHz for several 5 nm thick HEA thin films. d) Effective spin Hall conductivity obtained at 9 GHz versus thickness of three exemplary HEA thin films. e,f) Angular variation of *V*_S_ and *V*_A_ along with fits based on Equations (2) and (3) in the range between 0° and 360° for various 5 nm thick HEAs at *f =* 9 GHz.

To characterize the SOT generated by HEA thin films, we deposited a series of bilayers consisting of 5 nm HEA **|** 8 nm CoFeB, and fabricated µm‐sized devices (see Figure S5, Supporting Information) for spin‐torque ferromagnetic resonance (ST‐FMR) studies, which allows one to estimate θ_SH_ of the HEA films.^[^ ^19^, ^20^ ^]^ **Figure** **2**a shows ST‐FMR spectra of three different HEAs measured with a radio frequency (RF) current at 9 GHz in the presence of an in‐plane magnetic field that is swept between **+**0.2 and −0.2 T. The RF current applied to the HEA layer generates spin torques that give rise to the precession of the in‐plane magnetization of the CoFeB layer. The rectified dc voltage drop along the channel, *V*_mix_, arises from the anisotropic magnetoresistance of the CoFeB layer as the magnetization direction is changed via the SOT. A FMR signal thus results as the magnitude of the in plane magnetic field, *B*_ext_, is varied at a fixed angle.^[^ ^19^, ^20^ ^]^ The variation of *V*_mix_ with *B*_ext_ can be fitted by the equation:

(1) $$V_{mix}=V_{0}\;\;[V_{S}\;\frac{\Delta^{2}}{\Delta^{2}+{\left(B_{ext}-\;B_{res}\right)}^{2}}+V_{A}\;\frac{\Delta \;\left(B_{ext}-\;B_{res}\right)}{\Delta^{2}+{\left(B_{ext}-\;B_{res}\right)}^{2}}$$

where *V*_0_ is a pre‐factor, *V*_S_ and *V*_A_ are the symmetric and antisymmetric magnitudes of the Lorentzian response, *B*_res_ is the resonance field and Δ is the linewidth.^[^ ^32^ ^]^ The value of θ_SH_ can be extracted from the line‐shape profile of these measurements based on established models^[^ ^20^, ^33^, ^34^ ^]^ (see Supporting Information for a detailed analysis). We also note that the measured θ_SH_ does not account for the interface transparency between the HEA layer and the CoFeB layer, and, thus, it is to be considered as an effective θ_SH_, since there can be significant spin current dissipation at this interface.^[^ ^35^ ^]^

Figure 2b displays the variation of θ_SH_ as a function of the RF frequency *f*, for various HEAs. θ_SH_ varies slightly as a function of *f*. The values of θ_SH_ are high and decrease from ∼1 for IrRuAlGa, to ∼0.5 for IrCoRuAlGa, to ∼0.4 for IrCoNiRuAlGa, as shown in Figure 2c, as the number of elements in the alloy is increased from 4 to 6. Figure 2d shows the plot of the spin Hall conductivity (σ_SH_ **=** θ_SH_ **×** σ) for bilayer films with different HEA thicknesses (*t*). The value of σ_SH_ gradually decreases with increasing thickness of the HEA films, possibly correlated to the strain relaxation described earlier, although we noticed an increased variability of the σ_SH_ data for the IrRuAlGa films. The detailed thickness dependence of the ST‐FMR spectra and effective θ_SH_ for *t* HEA **|** 8 nm CoFeB thin films are shown in Figures S6–S8 (Supporting Information). Overall, we note that the SOT efficiency gradually decreases as we increase the number of elements in our HEA, thus diluting the amount of Ir, which has strong spin‐orbit coupling, from 31 at.%, to 24 at.%, and to 19 at.% respectively, while remaining high as compared to conventional SOT materials. This shows the highly attractive possibility of retaining efficient SOTs in HEAs even when the content of the heavy metal element is further reduced beyond that which we investigate here.

In order to clarify the direction of the spin‐orbit torques, we perform angle dependent ST‐FMR measurements by rotating the orientation of the micro‐strips with respect to the fixed magnetic field direction. We define the angle between the field direction and the current direction to be φ. The plots of *V*_S_ and *V*_A_ versus rotation angle φ can be fitted with the following equations by considering all possible damping‐like (τ_*DL*_) and field‐like (τ_*FL*_) spin‐orbit torques:^[^ ^36^, ^37^, ^38^ ^]^

(2) $$\;\;V_{S}\;\propto \;\tau_{DL,x}\sin\left(2\varphi \right)sin\left(\varphi \right)+\tau_{DL,y}\sin\left(2\varphi \right)cos\left(\varphi \right)+\tau_{FL,z}\sin\left(2\varphi \right)$$

(3) $$\;V_{A}\;\propto \;\tau_{FL,x}\sin\left(2\varphi \right)sin\left(\varphi \right)+\tau_{FL,y}\sin\left(2\varphi \right)cos\left(\varphi \right)+\tau_{DL,z}\sin\left(2\varphi \right)$$

The orientation of the damping‐like torques  τ_*DL*,*i*_ along the *i = x*,*y*,*z* directions are shown schematically in the insets to Figure 2e. The τ_*DL*,*y*_ derived from the spin current that is polarized along the *y* direction^[^ ^32^ ^]^ is much larger in comparison with the other damping‐like torques, as shown in Figure S10 (Supporting Information). Field‐like torques along the *y* direction (τ_*FL*,*y*_) make a large contribution to *V*_A_(φ), in comparison with the field‐like torques along the *x* direction (τ_*FL*,*x*_) and damping‐like torques along the *z* direction (τ_*DL*,*z*_), as shown in Figures 2f and S10 (Supporting Information).

**Figure 3:**
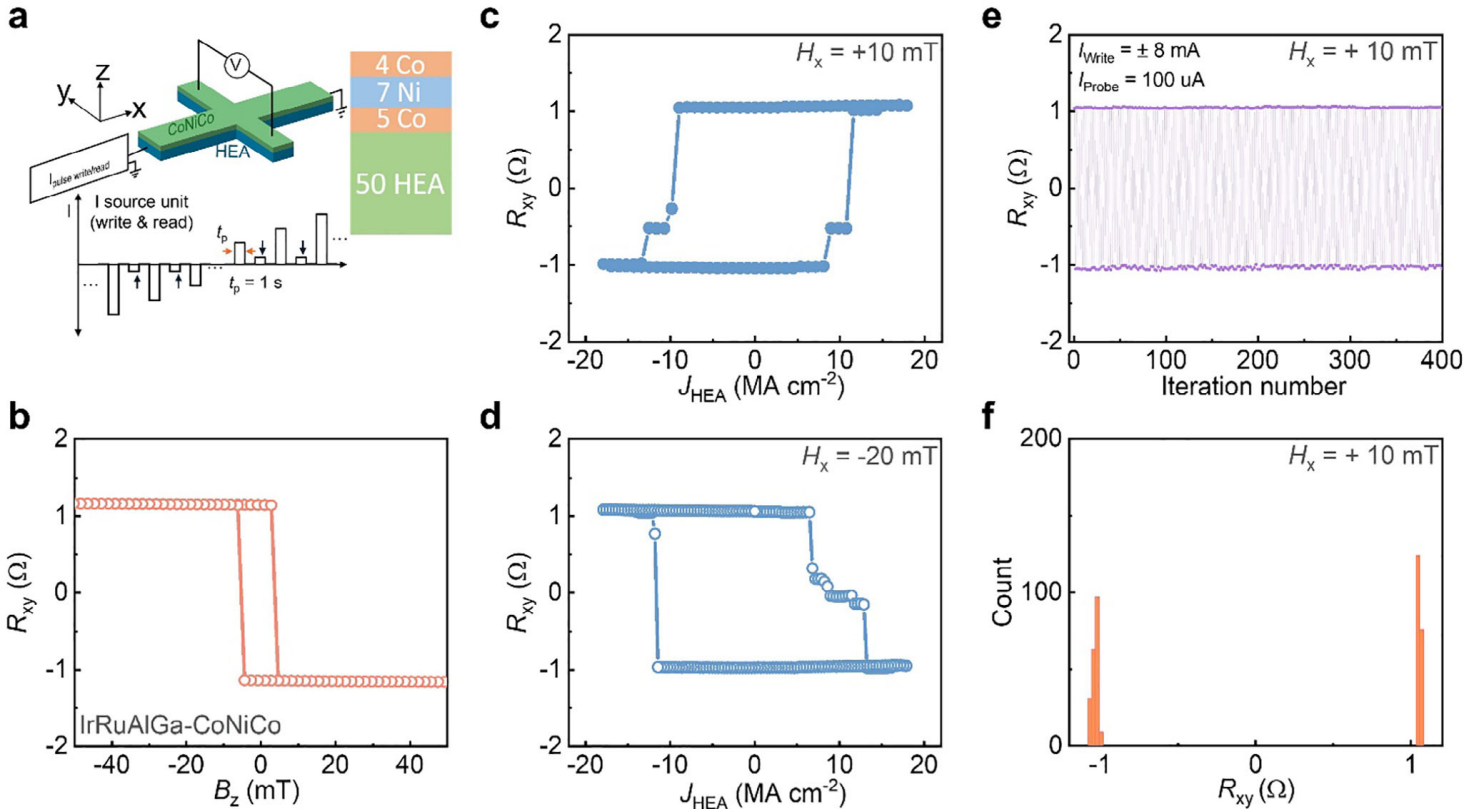
Current‐induced magnetization switching for Co | Ni | Co films grown on HEAs. a) Schematic illustration of the experimental setup and sequence of electrical input signals. b) Anomalous Hall resistance (*R*_xy_) as a function of out‐of‐plane magnetic field *B*_z_ for HEA = IrRuAlGa, measured at 300 K. c,d) Spin‐orbit torque induced magnetization switching with the assistance of an external in‐plane magnetic field of **+**10 and −20 mT, for a current pulse length of 1s. e) *R*_xy_ switched by a series of positive and negative current pulses as a function of iteration number with the assistance of an external in‐plane magnetic field of **+**10 mT. f) Histogram of data from e, demonstrating deterministic SOT switching of the device magnetization.

Spintronic devices often are fabricated from magnetic multilayered thin films with perpendicular magnetic anisotropy (PMA) and which take advantage of SOT for setting the magnetic state of the device.^[^ ^29^, ^30^, ^31^, ^39^ ^]^ To evaluate HEA thin films for such devices, we performed SOT magnetization switching experiments in a type‐z configuration^[^ ^39^ ^]^ based on Hall bar devices formed from 50 HEA **|** 5 Co **|** 7 Ni **|** 4 Co (all units are in Ångstrom) stacks, as shown in **Figures** **3**a and S12 (Supporting Information). The magnetization hysteresis loops for out‐of‐plane (Figure 5a) and in‐plane (Figure 5b) magnetic fields indicate that the Co **|** Ni **|** Co multilayers display robust PMA when grown on Ir‐based HEAs. From the magnetometry data we can calculate the perpendicular magnetic anisotropy energy, $K_{u}∼\mu_{0}M_{s}H_{k}/2$, where *M*_s_ is the saturation magnetization and *H*_k_ is the anisotropy field extracted from the in‐plane hysteresis loops. As shown in Figure S11 (Supporting Information), *K*_u_ gradually decreases as more elements are added to the HEA layer, due to the dilution of Ir which will thereby reduce the anisotropy induced at the HEA | Co interface.

The strong PMA of these devices is also reflected in their anomalous Hall resistance (*R*_xy_) versus magnetic field applied along the magnetic easy‐axis (out‐of‐plane). As shown in Figures 3b and S13 (Supporting Information), well‐defined square hysteresis loops are found. Current‐induced magnetization switching experiments for HEA = IrRuAlGa, IrCoRuAlGa, IrCoNiRuAlGa are shown in Figure 3, Figures S14,S15 (Supporting Information) respectively. Current pulses are applied along the Hall bar device channel in the presence of an external magnetic field, which is either parallel or antiparallel to the current direction, and a full magnetization switching is achieved at ≈10 MA cm^−2^. The polarity reversal upon the application of an opposite in‐plane magnetic field direction confirms the SOT origin of the switching of the magnetic layers (Figure 3c,d).^[^ ^34^ ^]^ Furthermore, we repeated the SOT switching experiments over 400 times, by alternating positive and negative current pulses, and a switching success rate of 100% was obtained, as shown in Figure 3e,f.

**Figure 4:**
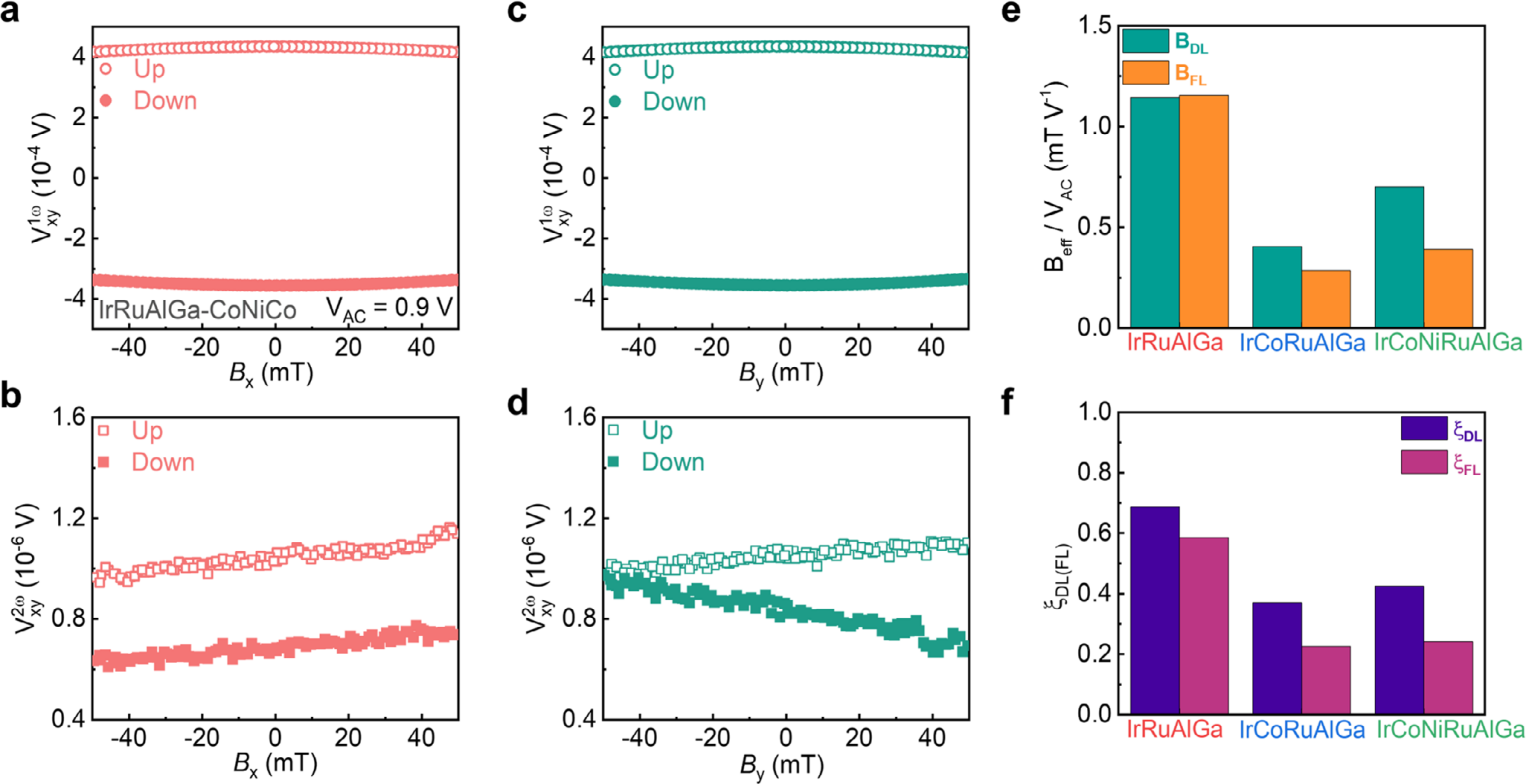
Harmonic Hall measurements for Hall bar devices formed from HEA | Co | Ni | Co thin film structure. a) $V_{\mathrm{xy}}^{1\omega }$ and b) $V_{\mathrm{xy}}^{2\omega }$, for HEA = IrRuAlGa, measured upon applying an alternating voltage (*V*_AC_) of 0.9 V, with the external magnetic field parallel to the direction of the applied current and c) $V_{\mathrm{xy}}^{1\omega }$ and d) $V_{\mathrm{xy}}^{2\omega }$ measured with the external magnetic field transverse to the direction of the applied current (Up and Down refer to the magnetization direction of the Co **|** Ni **|** Co film being positive or negative along *z*, as shown in Figure 3a). e) Damping‐like (*B*_DL_) and field‐like (*B*_FL_) components of the effective field per unit voltage, and f) damping‐like (*ξ*_DL_) and field‐like (*ξ*_DL_) components of the SOT efficiency for HEA thin films, after correction for the contribution of the planar Hall effect.

In addition, we performed harmonic Hall measurements to further quantify the magnitude of damping‐like and field‐like SOTs. **Figures** **4**a–d and S16–S18 (Supporting Information) show the first ($V_{\mathrm{xy}}^{1\omega }$) and second ($V_{\mathrm{xy}}^{2\omega }$) harmonic Hall voltages measured from Hall bar devices with different HEAs. An external magnetic field is swept along and transverse to the applied alternating voltage with a frequency of 117 Hz. We analyze the data using well‐known models for harmonic Hall measurements,^[^ ^40^, ^41^, ^42^, ^43^, ^44^, ^45^ ^]^ (see Supporting Information for detailed analysis) and quantify the damping‐like (*B*_DL_) and field‐like (*B*_FL_) components of the effective field, as well as the damping‐like (*ξ*_DL_) and field‐like (*ξ*_FL_) components of the SOT efficiency, as shown in Figure 4e,f respectively. The SOT efficiencies obtained by harmonic Hall measurements are in reasonable quantitative agreement, within the experimental error, with the ST‐FMR data shown in Figure 2, confirming the high SOTs generated by HEAs. Note that both ST‐FMR and harmonic Hall data clearly show that the SOT efficiency of our HEAs is at least two times higher than conventional SOT materials (e.g. Pt, Ta, W),^[^ ^15^, ^19^, ^20^ ^]^ for all the Ir contents that we considered.

**Figure 5:**
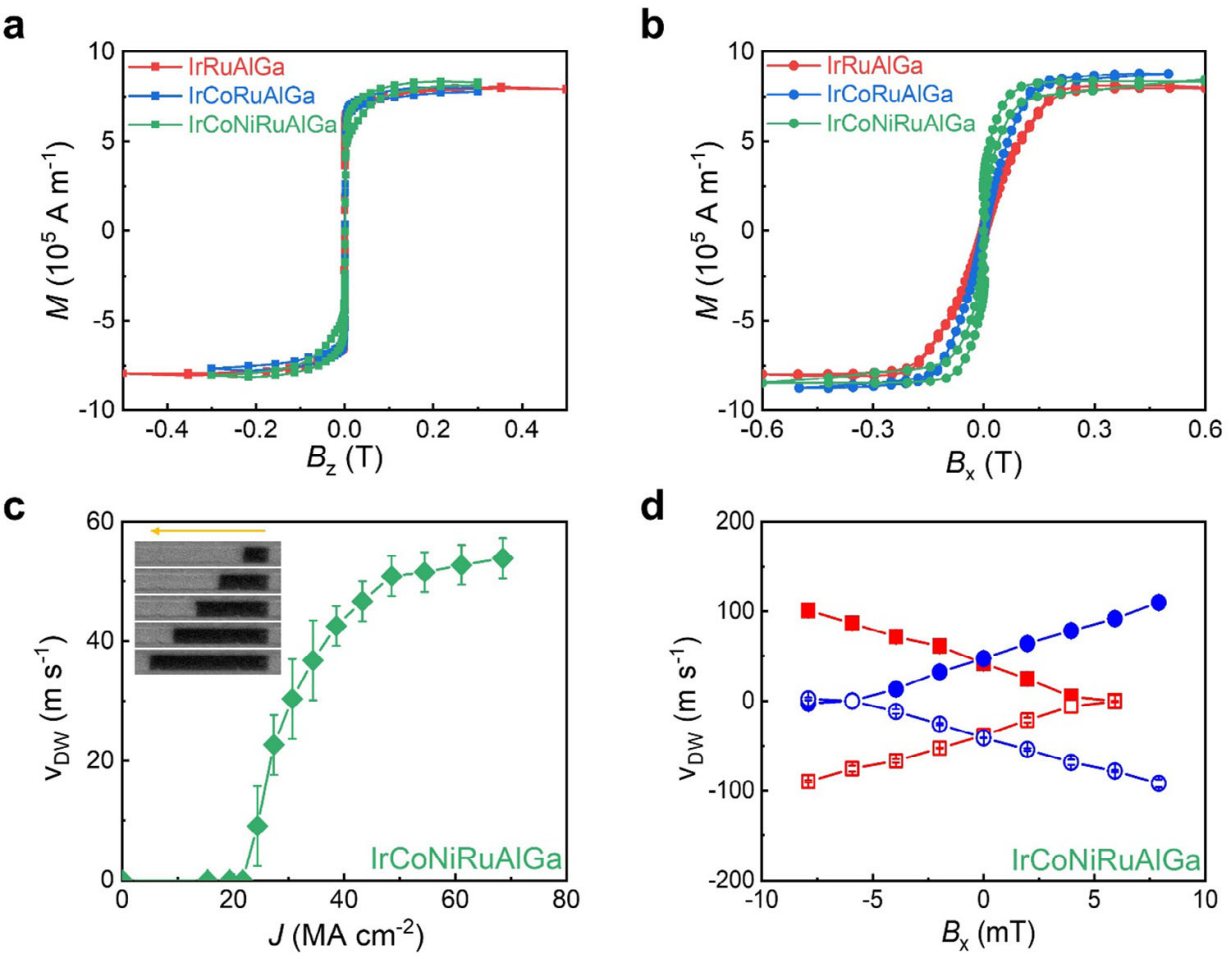
Magnetic properties and CIDWM of ferromagnetic Co | Ni | Co trilayers grown on top of HEA thin films. a,b) Out‐of‐plane and in‐plane magnetic hysteresis loops of 50 HEA | 5 Co | 7 Ni | 4 Co (units in Ångstrom, as shown in inset of Figure 3a), for 4‐, 5‐, and 6‐element HEA films. c) Domain wall velocity versus current density of ns‐long pulses sent along a racetrack fabricated from a 50 IrCoNiRuAlGa **|** 5 Co **|** 7 Ni **|** 4 Co thin film. Inset shows differential Kerr microscopy images of a 4 µm wide and 30 µm long racetrack during CIDWM measurements. d) Domain wall velocity versus magnetic field applied in the direction of the racetrack length. Solid and empty symbols correspond to positive and negative current density (60 MA cm^−2^). Red and blue symbol corresponds to ↑↓ and ↓↑ domain wall configurations respectively.

Thus, we have established that HEAs can generate considerable spin‐orbit torques via large spin Hall effects from ST‐FMR measurements, spin‐orbit torque induced magnetization switching and second harmonic Hall measurements. Finally, we investigate current‐induced domain wall motion (CIDWM) derived from HEA layers. To this end, we fabricated µm‐sized racetrack devices with an HEA layer formed from the 6‐element alloy, IrCoNiRuAlGa. We used Kerr microscopy to detect the domain wall velocity upon sending a series of short current pulses along the racetrack, as a function of the current density (**Figure** **5**c). In these devices, we find that the domain walls can be displaced smoothly and reliably by ns‐long current pulses along the entirety of the racetrack device (see inset to Figure 5c). The domain walls (DWs) move at a relatively low speed, but nevertheless at a current density which is three times smaller for the same DW speed in similar devices with conventional SOT layers made of Pt.^[^ ^26^, ^27^ ^]^ To investigate the origin of the low domain wall velocity, *v*_DW_, we have performed CIDWM measurements in the presence of an external field aligned in the direction of the racetrack (Figure 5d). By plotting *v*_DW_ vs the applied field, the effective DMI field, *H*_DMI_, can be extracted,^[^ ^26^ ^]^ which here is ∼6 mT. This value is quite small compared to conventional materials for racetracks, and thus explains the limited velocity reached by the DWs for these HEAs. The relatively low values of *H*_DMI_ are consistent with the lowered values of *K*_u_ mentioned above due to the reduced Ir content in the HEA layers. Note that for some spintronic applications low DW velocities are sufficient e.g. for racetrack memory as a successor to V‐NAND flash memories.

**Figure 6:**
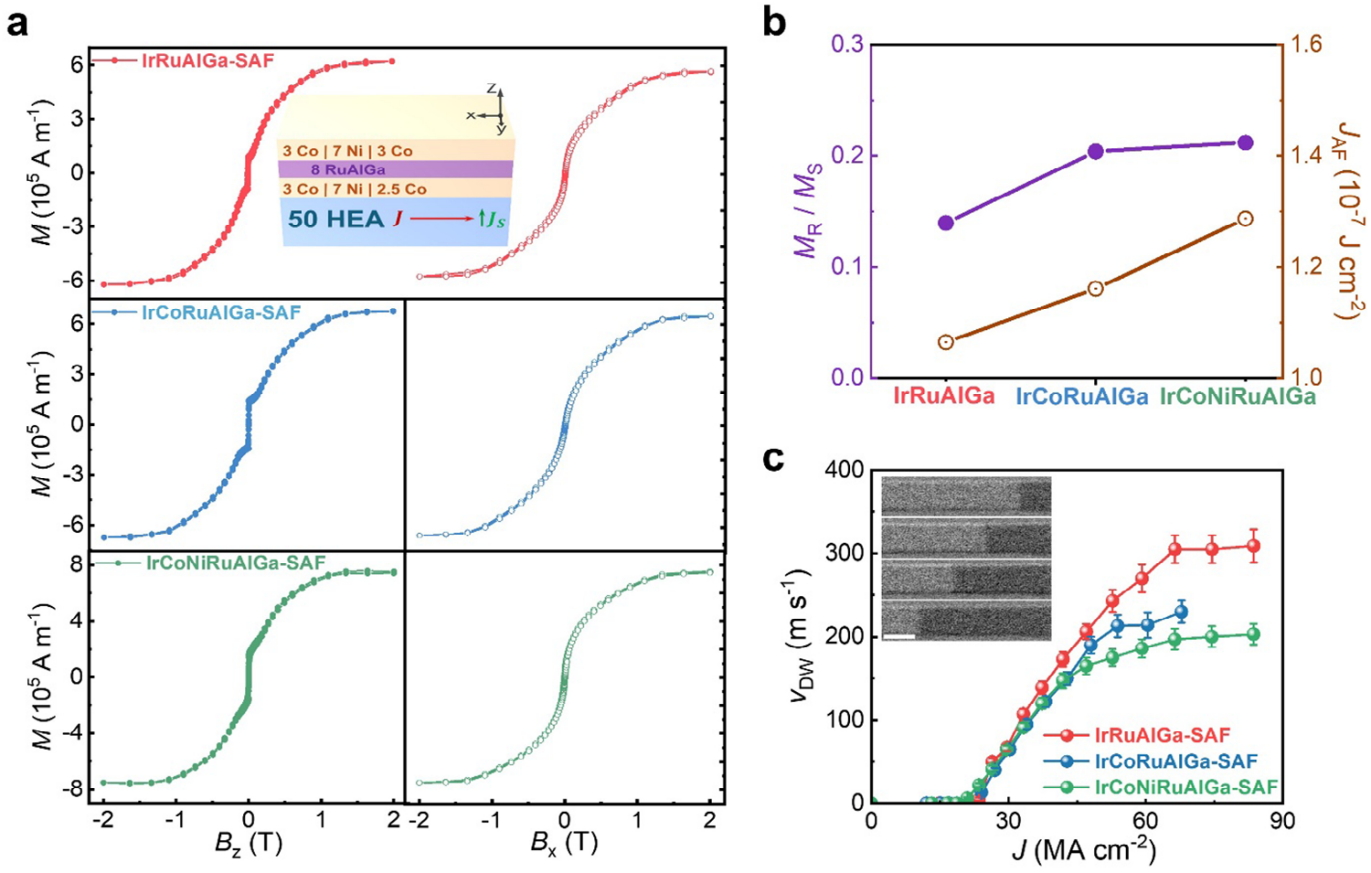
Magnetization properties and current‐induced motion of magnetic domain walls for HEA | SAF racetracks. a) Out‐of‐plane (*B*_z_) and in‐plane (*B*_x_) magnetic hysteresis loops of HEA | SAF films (inset: detailed film structure). b) *M*_R_ **/** *M*_S_ ratio and antiferromagnetic exchange coupling strength (*J*_AF_) for SAF films grown on serveral HEAs. c) Domain wall velocity (*v*) versus current density in µm‐sized racetracks formed from various HEA **|** SAF thin films. Inset shows differential Kerr microscopy images of an IrCoNiRuAlGa‐SAF racetrack taken sequentially after injecting 6 current pulses (50 MA cm^−2^). The scale bar in the inset corresponds to 5 µm.

The most interesting DW based devices are those formed from synthetic antiferromagnets (SAFs)^[^ ^46^ ^]^ in which it has been shown that SOTs can move DWs very efficiently and up to very high speeds. Here, we use a ternary RuAlGa alloy as a novel antiferromagnetic (AF) coupling layer (with a thickness of ∼0.8 nm) to antiferromagnetically exchange couple an upper (3 Co **|** 7 Ni **|** 3 Co) and a lower (3 Co **|** 7 Ni **|** 2.5 Co) ferromagnetic layer (bottom to top, units in Ångstrom). Note that the tetragonal structure of RuAlGa is key to realize SAFs with tetragonal Ir‐based HEAs as spin Hall layers (inset to **Figure** **6**a). By introducing SAFs based on RuAlGa, we can not only reduce the amount of the heavy metal Ru, which is conventionally used as an AF coupling layer in spintronic applications, but we also enable a higher degree of tunability of the lattice parameter by varying the composition of RuAlGa, which is key for the development of novel spintronic heterostructures.^[^ ^21^ ^]^

The magnetic hysteresis loops of HEA **|** SAF samples displayed in Figure 6a show an out‐of‐plane spin‐flop transition, thereby, proving that a RuAlGa spacer layer induces strong antiferromagnetic coupling between the top and bottom Co **|** Ni **|** Co layers. The ratio of remanent magnetization (*M*_R_) to saturation magnetization (*M*_S_) shown in Figure 6b is measured to be ≈0.1–0.2, which is close to the ideal value of zero for a SAF. Note that *M*_R_ **/** *M*_S_ gradually increases with increasing number of elements in the HEA layer, which is very likely related to a reduction of the proximity‐induced magnetization in the HEA layer due to the dilution of the Ir content.^[^ ^27^ ^]^ Furthermore, the in‐plane magnetic hysteresis loops from Figure 6a show a large antiferromagnetic exchange coupling field (*B*_ex_) between the top and bottom Co **|** Ni **|** Co layers which is close to 1.8 T. Such a strong exchange coupling can provide a significant exchange coupling torque to induce fast current‐induced motion of the magnetic domain walls.^[^ ^46^ ^]^ Furthermore, the resulting antiferromagnetic exchange coupling strength (*J*_AF_)^[^ ^47^ ^]^ is enhanced by introducing more elements into the HEA thin films, which shows a correlation with the out‐of‐plane lattice parameter of the 5 nm thick HEA films (Figure 6b).

We investigate the CIDWM in these SAF films by Kerr microscopy measurements of µm‐sized racetrack devices and extract the dependence of the domain wall velocity (*v*) versus current density (*J*) flowing through the racetrack (Figure 6c). Notably, in these HEA **|** SAF structures we find fast domain wall motion with speeds up to 300 m s^−1^, at a current density of only 65 MA cm^−2^, i.e., three times lower than for conventional Pt | SAF films.^[^ ^46^ ^]^ We also find that the domain walls can be reliably moved back and forth along the entire length of the racetracks (i.e., 30 µm), which further confirms the quality and uniformity of the deposited HEA **|** SAF films (see Figure S19, Supporting Information). The maximum *v*_DW_ gradually decreases with increasing number of elements in the HEA layer. We find that this reduction is, in large part, due to changes in *M*_R_ **/** *M*_S_, as shown in Figure 6a. Thus, we anticipate that the domain wall velocity will reach even higher values for HEA‐based SAFs with perfect magnetic moment compensation.^[^ ^46^ ^]^ Note that here the high domain wall velocities are the result of the strong exchange coupling torque given by antiferromagnetic coupling from the RuAlGa layer, which plays a dominant role, as compared to the reduced *H*_DMI_ in the simpler HEA **|** Co **|** Ni **|** Co structures.^[^ ^26^, ^27^, ^46^ ^]^ At the same time, the HEA **|** SAF stacks retain highly‐efficient domain wall motion at low current densities, with the same threshold current density for HEAs with different number of elements (Figure 6c), indicating that the significant dilution of Ir in HEAs does not undermine the SOT efficiency in domain‐wall‐based spintronic devices. We speculate that this could be related to an increased interface transparency for the spin current, which plays a critical role in the SOT efficiency.^[^ ^35^ ^]^

## 3 Conclusion

In conclusion, we have demonstrated the successful growth of iridium based high entropy alloy thin films with an alternating layered tetragonal crystal structure by magnetron sputter deposition techniques at ambient temperature. These HEA thin films can efficiently convert charge current into spin current, despite a significant dilution of the Ir content as the number of elements is increased. Multilayered thin films of cobalt and nickel can be grown epitaxially on these HEAs, showing perpendicular magnetic anisotropy and domain wall motion at low current densities. We have also successfully deposited synthetic antiferromagnets on HEA layers using a novel multi‐element RuAlGa spacer layer as the exchange coupling layer. Finally, we show that the spin‐orbit torques generated by Ir based HEAs can efficiently drive magnetic domain walls in epitaxially‐engineered SAFs at high speeds and at low current densities. The HEAs with reduced heavy metal content investigated here are a promising family of candidates for spin‐orbit torque generation, with vast potential for superior performance and characteristics for spintronic devices.

## 4 Experimental Section

### Thin Films Growth and Characterization

The films were deposited in an AJA ‘Flagship Series’ sputtering system in the presence of Ar gas on 10 **× **10 mm^2^ MgO substrates with a [001] orientation. The base pressure before deposition was less than 1.33 **×** 10^−6^ Pa and the argon pressure during deposition was 0.4 Pa. The high entropy alloy thin films and the ternary alloy RuAlGa were prepared by co‐sputtering at ambient temperature from individual metal targets including Co, Ni, Ir, Al, Ru, Ir_30_Ga_70_ and Ru_35_Ga_65_ targets with a 2‐inch diameter and 0.25 inch thickness. A capping layer of MgO was grown by off‐axis radio‐frequency sputtering. The CoFeB layer was prepared by sputtering from a single target with a nominal composition of Co_20_Fe_60_B_20_. The composition of the thin films was determined by non‐destructive Rutherford Backscattering spectroscopy with an accuracy of ∼1–2 at.%. The atomic ratios of the RuAlGa, IrRuAlGa, IrCoRuAlGa, and IrCoNiRuAlGa films were found to be 46:23:31, 31:15:23:31, 24:25:8:18:25, and 19:21:15:7:16:22, respectively. High‐resolution XRD measurements were performed at room temperature using a Bruker D8 Discover system with Cu‐*K*α_1_ radiation (*λ* **=** 1.54 Å). Magnetization hysteresis loops were measured using a superconducting quantum interference device vibrating sample magnetometer (SQUID‐VSM). Lamellas were prepared for cross‐section TEM by focused ion‐beam milling using a Tescan GAIA3 instrument. TEM data were obtained with a JEOL ARM300F2 transmission electron microscope. The primary electron energy was 300 kV. Device fabrication was carried out using optical lithography and ion‐beam etching. Subsequently, 3 nm thick Ti and 80 nm thick Au electrical contact layers were deposited using a lift‐off process. The resistance of the HEA films were measured using a Veeco FPP5000 four‐point probe station.

### Spin‐Torque Ferromagnetic Resonance Measurements

ST‐FMR measurements were performed at room temperature in micro‐strip devices of various sizes. A gigahertz frequency probe tip (Picoprobe Model 40A) was used to inject an RF excitation current (Keysight MXG N5183B signal generator) at a power of 20 dBm. The rectified DC voltage across the micro‐strip was simultaneously measured using a bias‐tee (Tektronix PSPL5545) and a nanovoltmeter (Keithley 2182A). The rectified DC voltage resulting from mixing of the RF current with the varying resistance of the micro‐strip due to the anisotropic magnetoresistance of the Co_20_Fe_60_B_20_ layer gives rise to the FMR signal. The FMR signal at a particular excitation frequency is measured as the external in‐plane magnetic field is swept, at an angle of 45° to the long axis of the micro‐strip. The effective spin Hall angle is obtained by extraction of the ferromagnetic resonance curve shape peak. For ST‐FMR measurements, the data from 5 devices for each film are averaged, with the error bars corresponding to one standard deviation.

### Electrical Transport Measurements

Electrical transport measurements, i.e., anomalous Hall and harmonic Hall measurements, were performed at room temperature using: current source: Kethley 6221, voltmeter: Keithley 2182a, lock‐in amplifier: Zurich instruments MFLI 500 kHz. For current‐induced magnetization switching, a current source (Keithley 6221) was programmed to deliver 1 s long write current pulses (sweeping from −10 to +10 mA in 0.5 mA steps) with a read current of 0.1 mA in between the write current pulses. Note that it is confirmed that 0.1 mA does not affect the current‐induced magnetization switching (switching current ∼8 mA).

### Current‐Induced Domain Wall Motion Measurements

Magneto‐optical Kerr effect microscopy in differential mode was used to monitor the position of DWs along the µm‐sized racetrack devices, using a modified EVICO system, in which the devices were wire bonded and connected via an RF line to a voltage pulse generator (PSPL10300B). Images are taken after a fixed number of 5 or 10 ns long current pulses are sent along the racetrack device, chosen such that the DW has moved by a significant distance, typically several micrometers. The DW velocity was then determined, assuming that the DW moves only during the current pulses, as the ratio between the DW displacement detected by differential Kerr microscopy and the total length of the current pulses. The error bar for the velocity in the CIDWM measurement was calculated as the ratio between the resolution limit of the Kerr microscope, which is estimated to be 1 µm, and the total pulse duration. The measurements of the DW velocity as a function of the applied magnetic field were repeated 3 times for each data point, with the error bar corresponding to one standard deviation.

## Conflict of Interest

The authors declare no conflict of interest.

## Author Contributions

P.W. and A.M. contributed equally to this work. P.W. conceived the project. P.W. designed the thin film stacks, carried out thin film growth, characterization, device fabrication, and magneto‐transport measurements. A.M. conducted domain wall motion measurements. J.‐C.J. and Y.‐C.L. performed current induced magnetization switching and harmonic Hall measurements. I.K. conducted all RBS calibration of the thin film samples. H.D. carried out HR‐TEM imaging. P.W., A.M. and S.S.P.P wrote the manuscript. All authors discussed the data and commented on the manuscript. S.S.P.P. supervised the project.

## Supporting information



Supporting Information

## Data Availability

The data that support the findings of this study are available from the corresponding author upon reasonable request.
