# Supplementary material for: High Entropy Alloy Thin Films as Efficient Spin‐Orbit Torque Sources for Spintronic Memories
Source: Adv Mater. 2025 Jun 4;37(30):2416820. doi: 10.1002/adma.202416820 (PMC12306383; doi:10.1002/adma.202416820)
Supplement: Supplementary file 1 — Supporting Information [file ADMA-37-2416820-s001.docx]

Supporting Information for

**High Entropy Alloy Thin Films as Efficient Spin-Orbit Torque Sources for Spintronic Memories**

Peng Wang^1^, Andrea Migliorini^1^, Yung-Cheng Li^1,2^, Hakan Deniz^1^, Ilya Kostanovski^1^, Jae-Chun Jeon^1^, Stuart S. P. Parkin^1,2*^

^1^Max Planck Institute of Microstructure Physics, 06120 Halle (Saale), Germany

^2^Institute of Physics, Martin Luther University Halle-Wittenberg, 06120 Halle (Saale), Germany

*e-mail: [stuart.parkin@mpi-halle.mpg.de](mailto:stuart.parkin@mpi-halle.mpg.de)

**The PDF file includes:**

Supplementary Text

**Figure** **S1** to **S25**


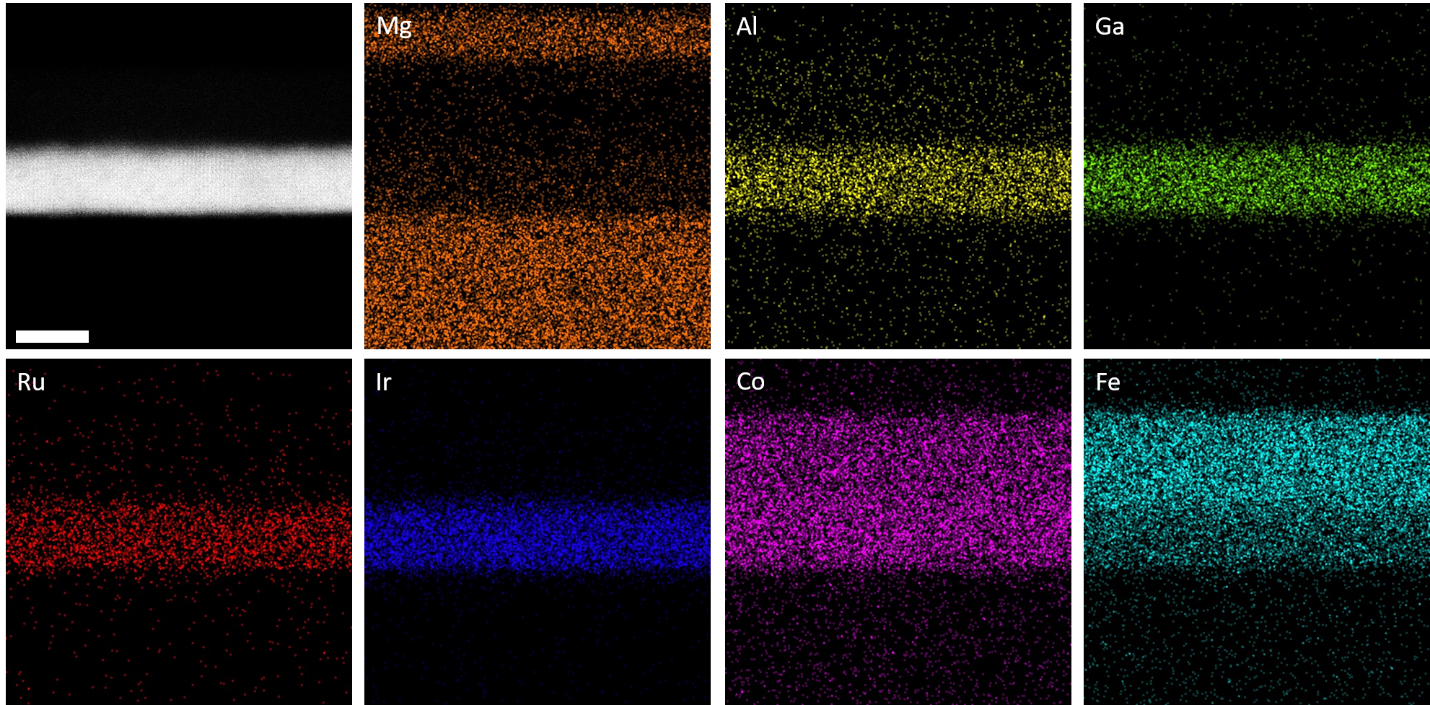


**Figure S1. Energy dispersive X-ray spectroscopy elements mapping for 5 nm Ir_31_Ru_15_Al_23_Ga_31_ | 8 nm Co_20_Fe_60_B_20_ thin film.** The Co and Fe signal at the HEA film position is an EDX artefact due to the scattering by the heavy element Ir. The scale bar corresponds to 5 nm.


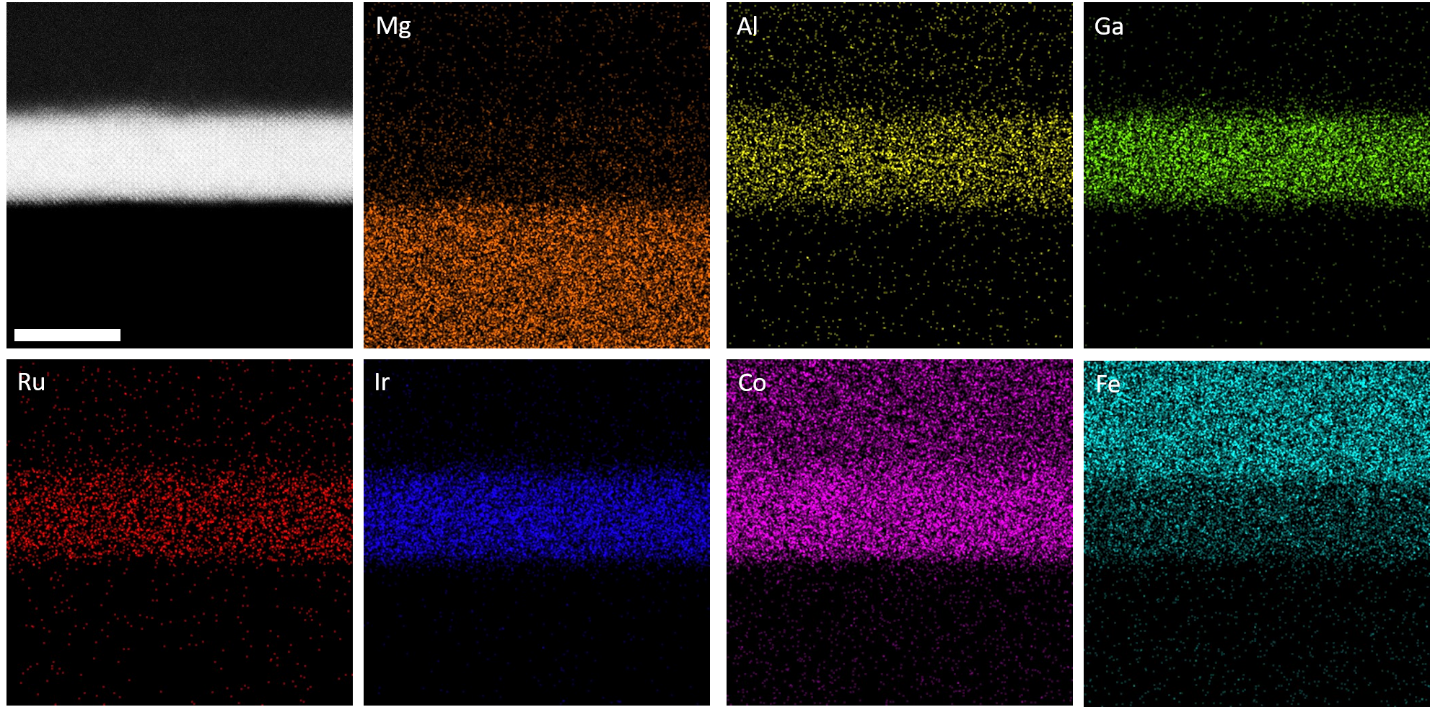


**Figure S2. Energy dispersive X-ray spectroscopy elements mapping for 5 nm Ir_22_Co_28_Ru_8_Al_17_Ga_25_ | 8 nm Co_20_Fe_60_B_20_ thin film.** The Fe signal at the HEA film position is an EDX artefact due to the scattering by the heavy element Ir. The scale bar corresponds to 5 nm.

**
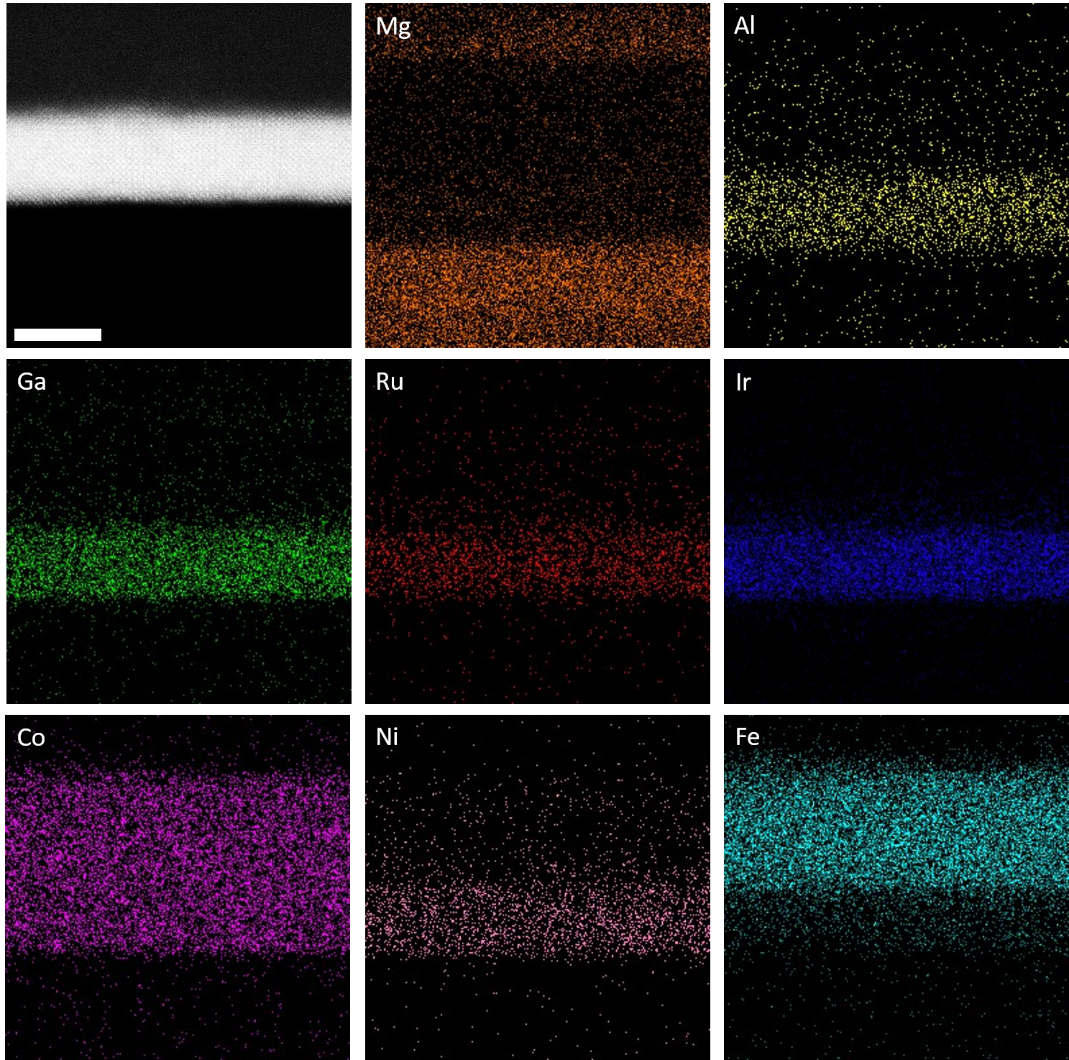
**

**Figure S3. Energy dispersive X-ray spectroscopy elements mapping for 5 nm Ir_19_Co_21_Ni_15_Ru_7_Al_16_Ga_22_ | 8 nm Co_20_Fe_60_B_20_ thin film.** The Fe signal at the HEA film position is an EDX artefact due to the scattering by the heavy element Ir. The scale bar corresponds to 5 nm.


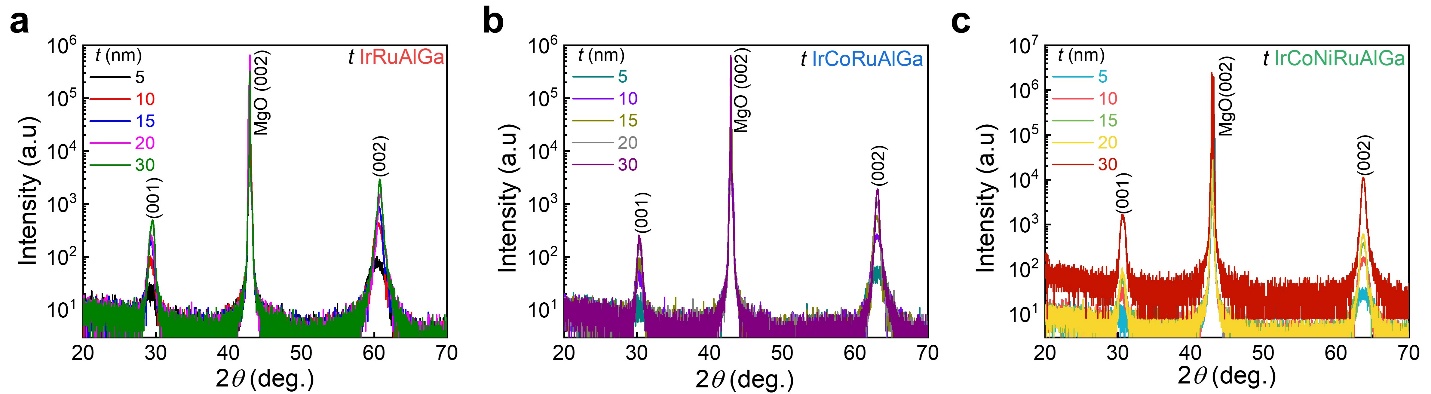


**Figure S4. Crystal structure of thickness dependent iridium based high entropy alloy thin films. a-c**) Thickness dependent 2$\theta$ - *ω* XRD specular scans for three different high entropy alloy thin films whose composition is given in the figure.


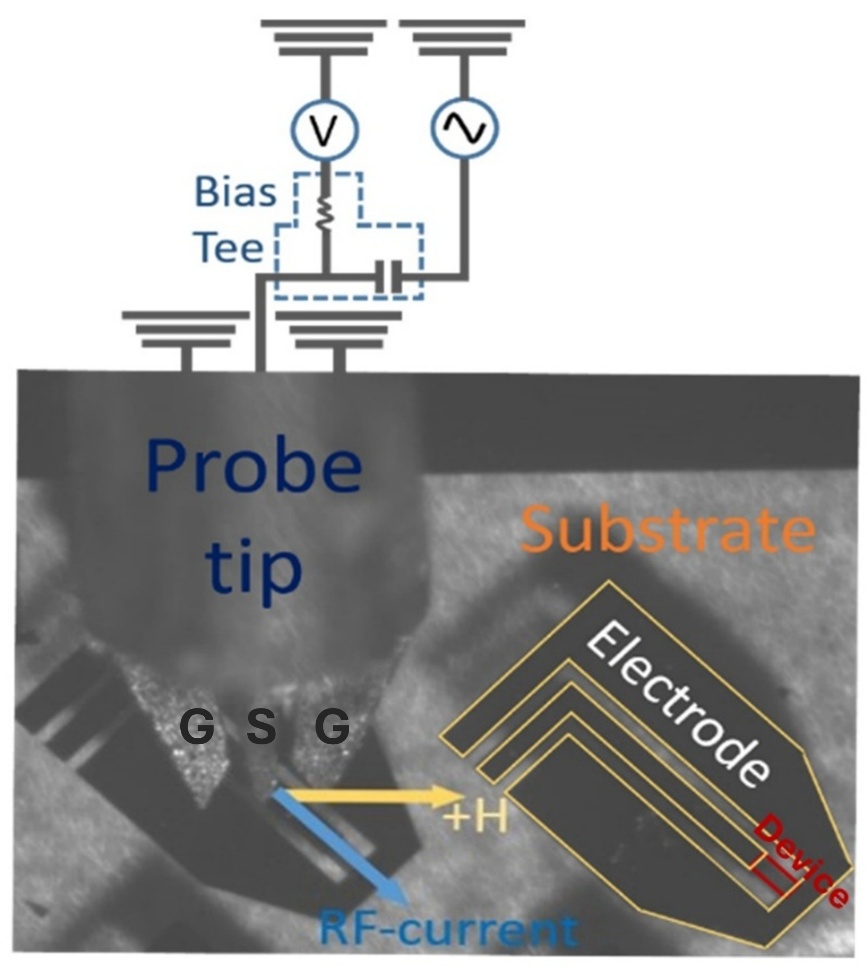


**Figure S5. Illustration of ST-FMR measurement setup using a ground-source-ground (G-S-G) probe tip.** The device consists of a channel that is 75 microns long and 37.5 microns wide, indicated by a red rectangle, while the electrical contacts are indicated by yellow lines.


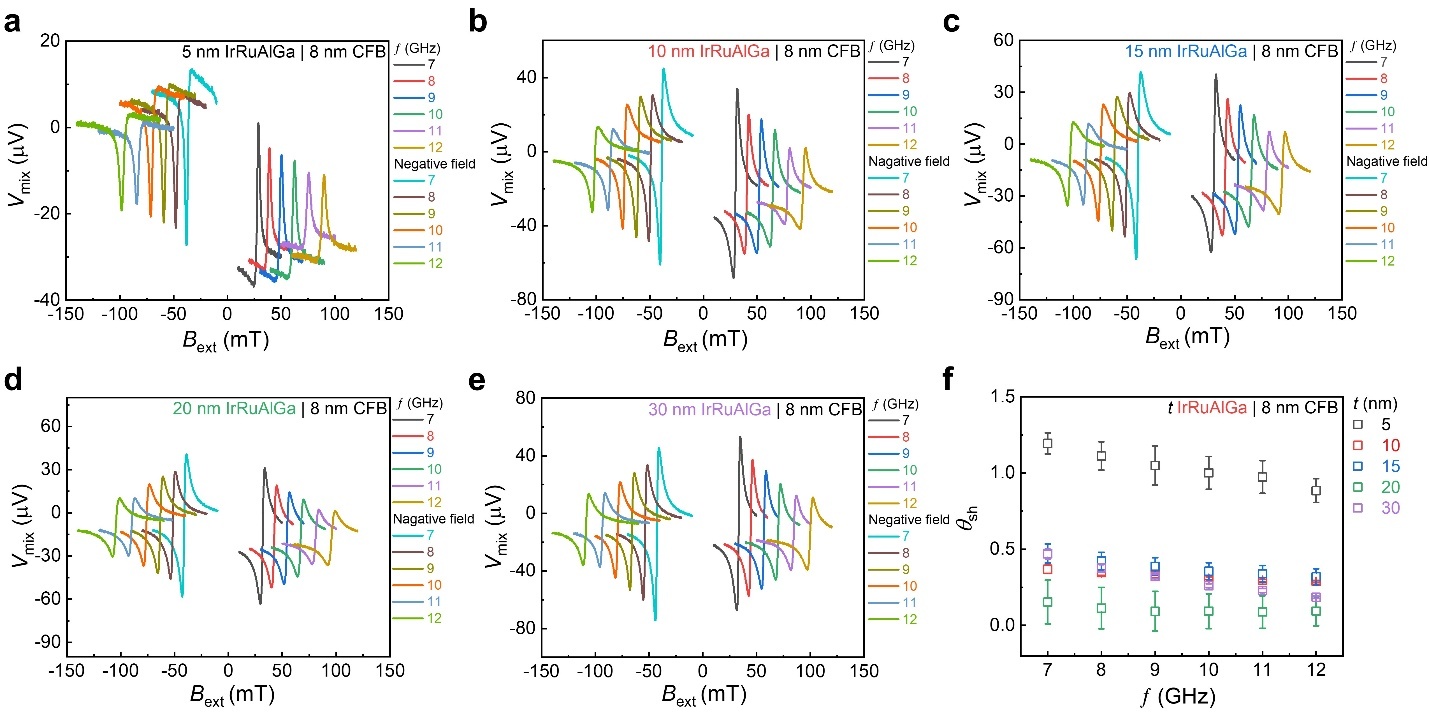


**Figure S6. Spin-torque ferromagnetic resonance measurements for bilayer thin films consisting of *t* IrRuAlGa | 8 nm Co_20_Fe_60_B_20_. a**-**e**) Frequency dependent ST-FMR spectra measured for devices with variable *t* from 5 to 30 nm. *t* is given in each figure. **f**) Effective spin Hall angle as a function of *f* from 7 to 12 GHz for *t* varying from 5 to 30 nm.


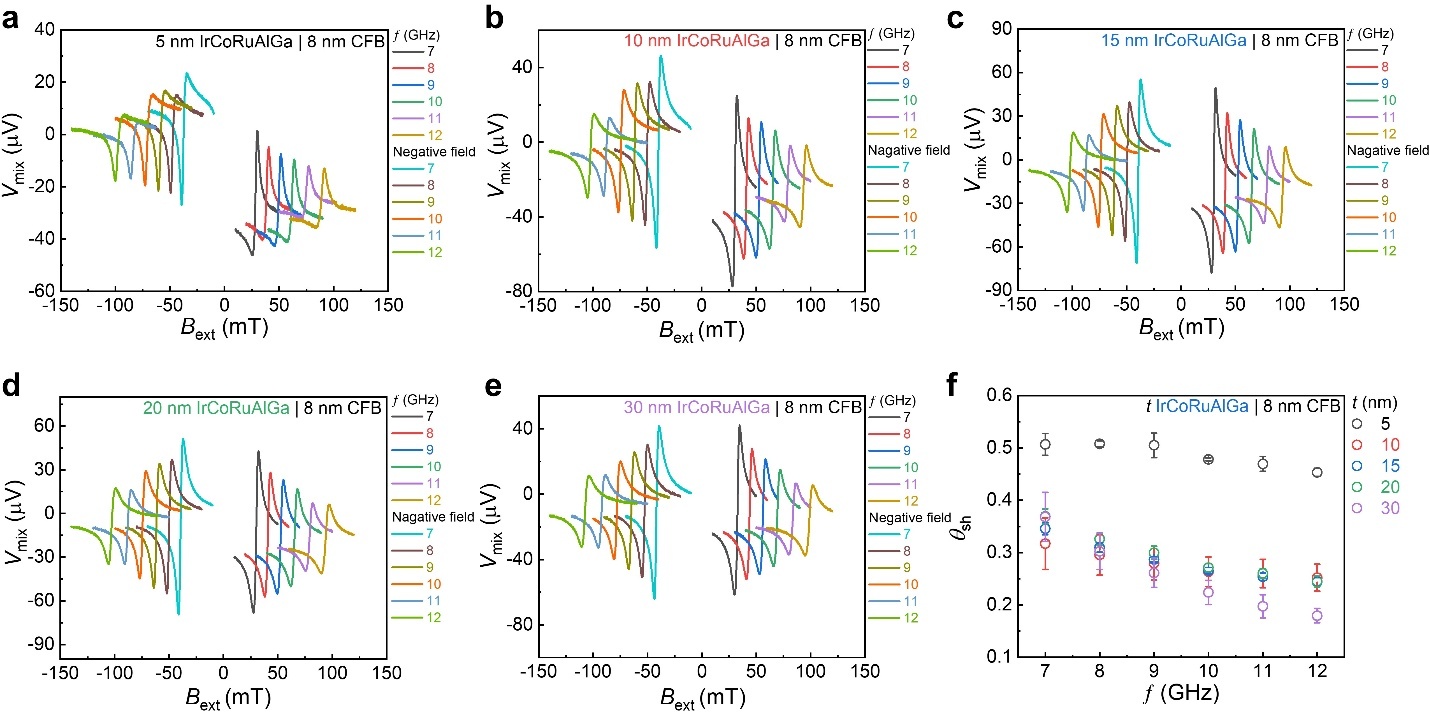


**Figure S7. Spin-torque ferromagnetic resonance measurements in bilayer thin films consisting of *t* IrCoRuAlGa | 8 nm Co_20_Fe_60_B_20_. a**-**e**) Frequency dependent ST-FMR spectra for *t* varying from 5 to 30 nm. **f**) Effective spin Hall angle as a function of *f* from 7 to 12 GHz for *t* varying from 5 to 30 nm.


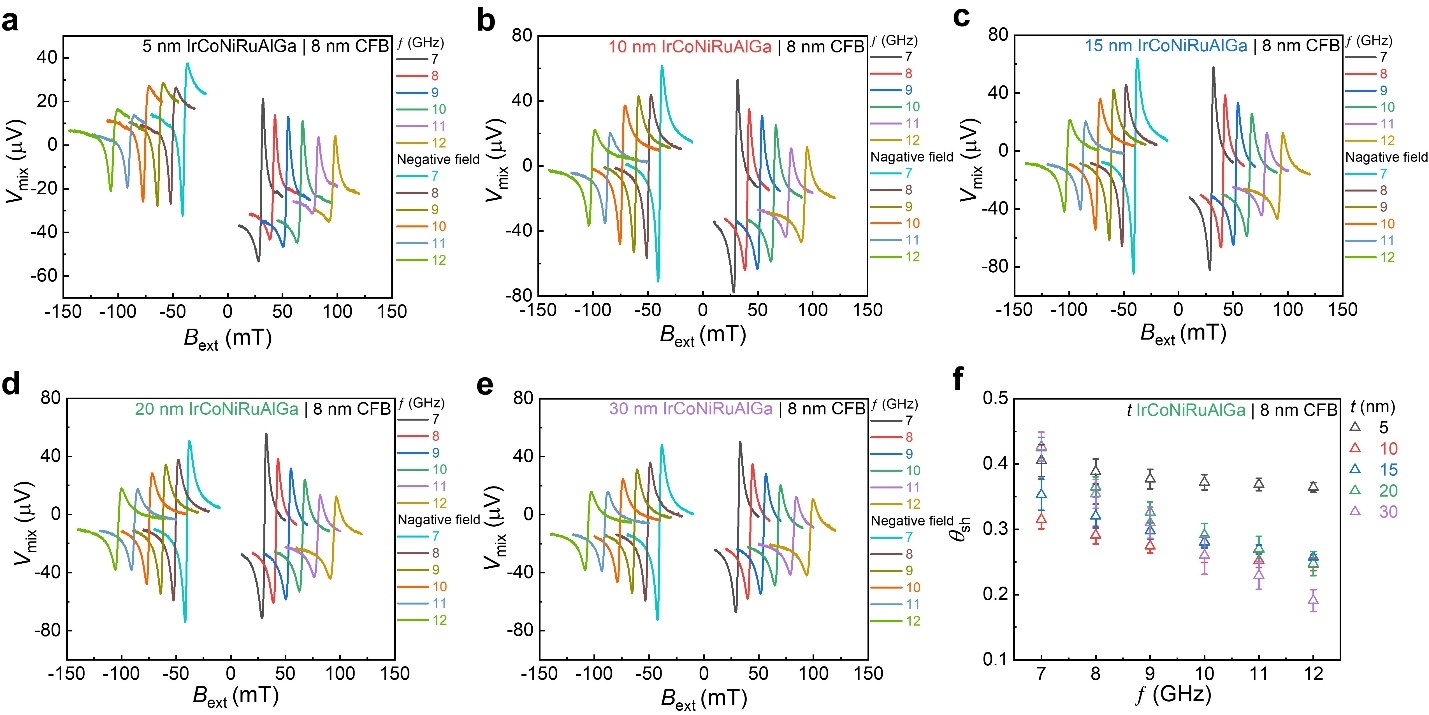


**Figure S8. Spin torque ferromagnetic resonance measurements in bilayer thin films consisting of *t* IrCoNiRuAlGa | 8 nm Co_20_Fe_60_B_20_. a-e**) Frequency dependent ST-FMR spectra measured from *t* varying from 5 to 30 nm. **f**) Effective spin Hall angle as a function of *f* from 7 to 12 GHz for *t* varying from 5 to 30 nm.


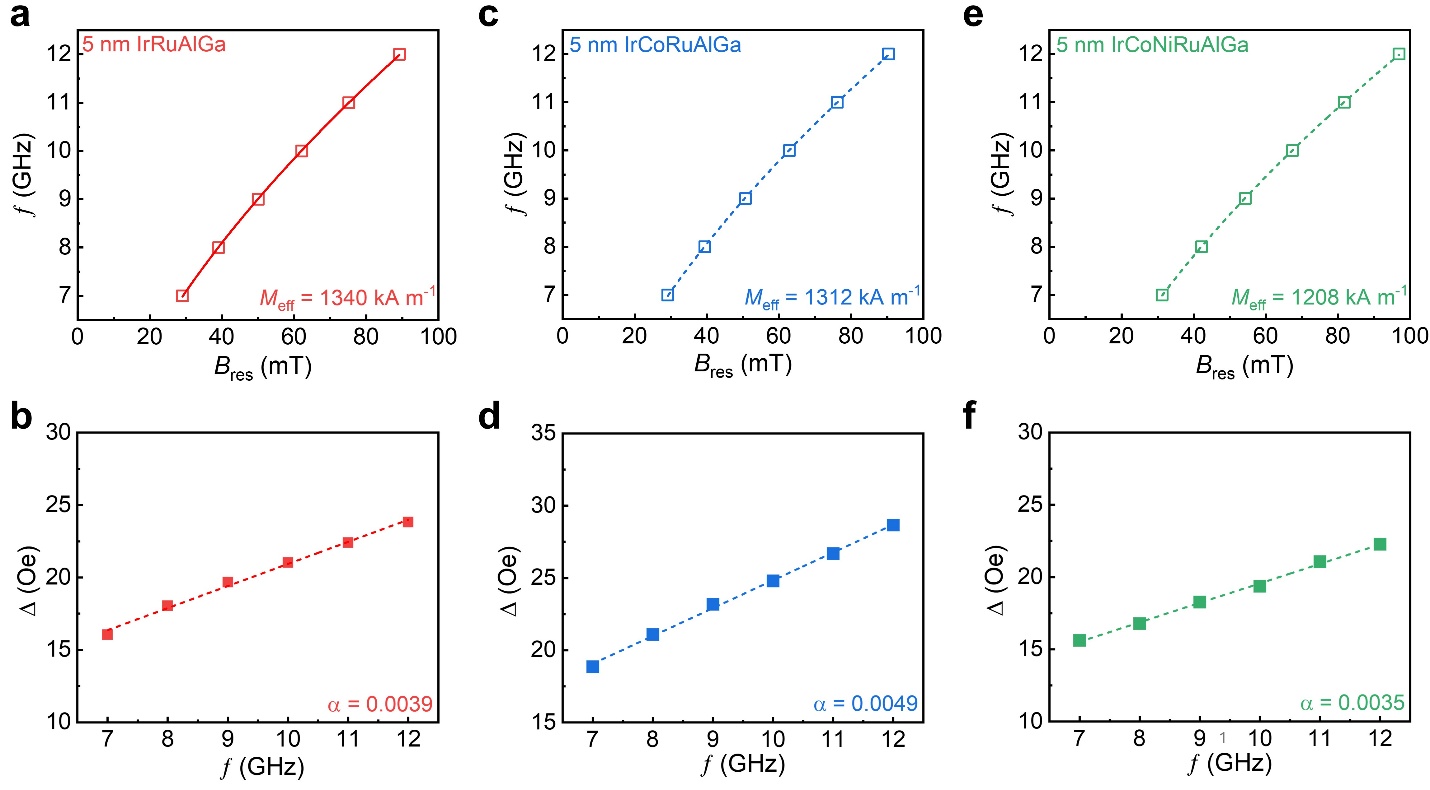


**Figure S9. a**,**c**,**e**) Resonant frequency as a function of the resonant field for 5 nm HEA **|** 8 nm Co_20_Fe_60_B_20_ samples used in the Kittel formula fit. The effective demagnetization field for each sample is given in each figure. **b**,**d**,**f**) Change of linewidth as a function of frequency for 5 nm HEA **|** 8 nm Co_20_Fe_60_B_20_ samples. The Gilbert damping constant is indicated in each figure.


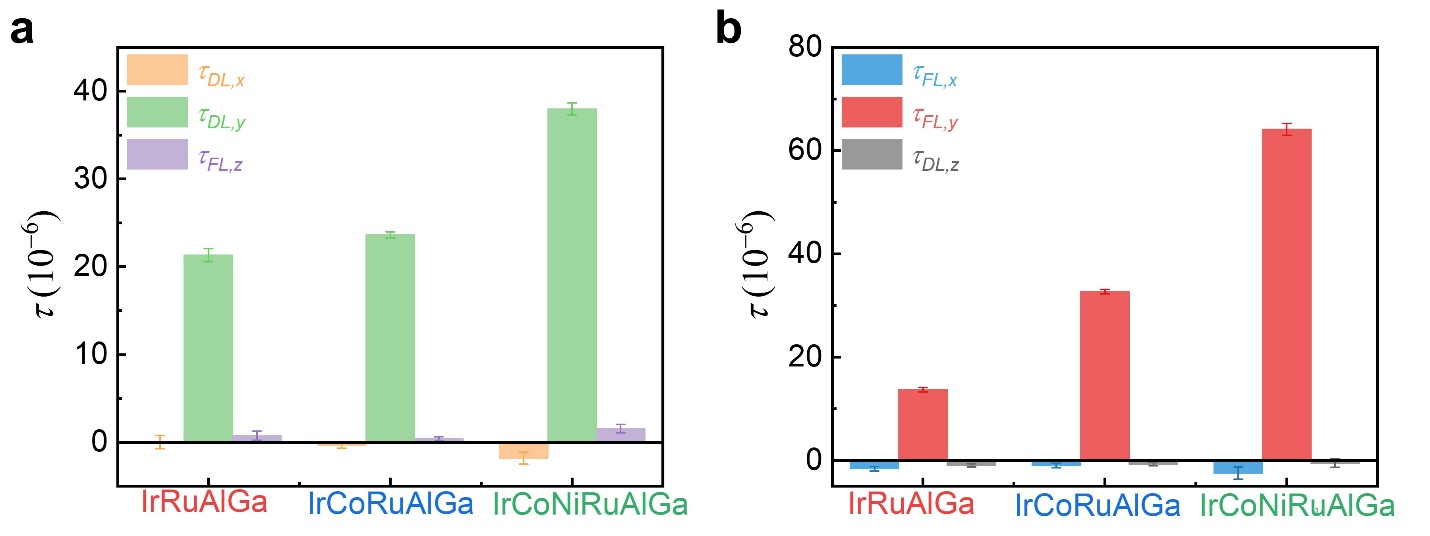


**Figure S10. Spin**-**orbit torques in different directions measured for several 5 nm HEA | 8 nm Co_20_Fe_60_B_20_ bilayer films.** **a**) Summary of damping-like torques ($\tau$_DL_) along *x*, *y* direction and field-like torque ($\tau$_FL_) along the *z* direction for different HEAs. **b**) Summary of field-like torques along *x*, *y* direction and damping-like torque along *z* direction for different HEAs.

**
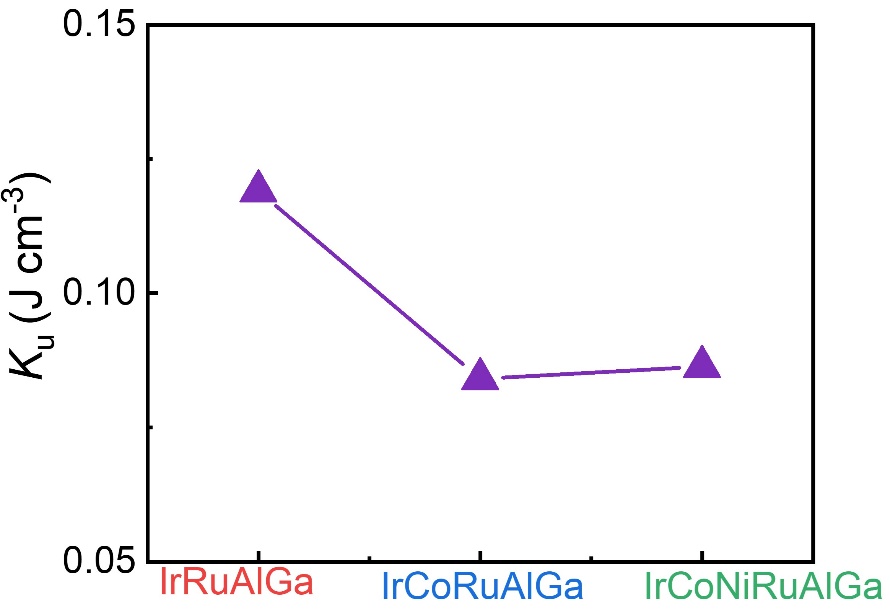
**

**Figure S11.** Magnetic anisotropy energy of films with structure 50 HEA **|** 5 Co **|** 7 Ni **|** 4 Co (all units are in Ångstrom), for different HEAs.


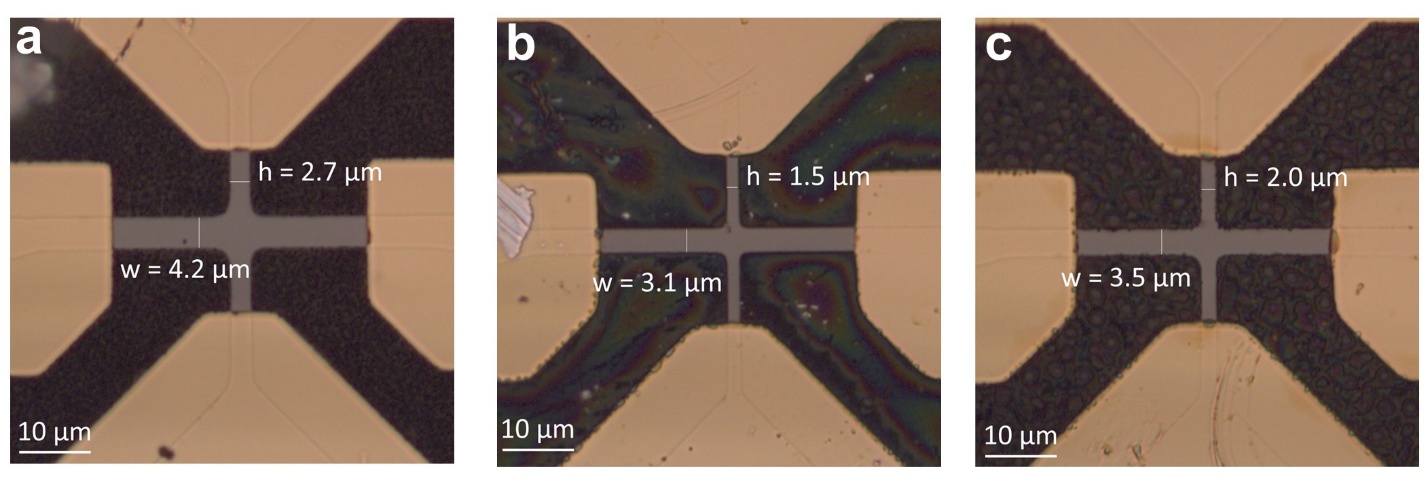


**Figure S12.** Optical images of μm-sized Hall bar devices made from films with structure 50 HEA **|** 5 Co **|** 7 Ni **|** 4 Co (all units are in Ångstrom), where HEA corresponds to **a**) IrRuAlGa, **b**) IrCoRuAlGa, and **c**) IrCoNiRuAlGa.


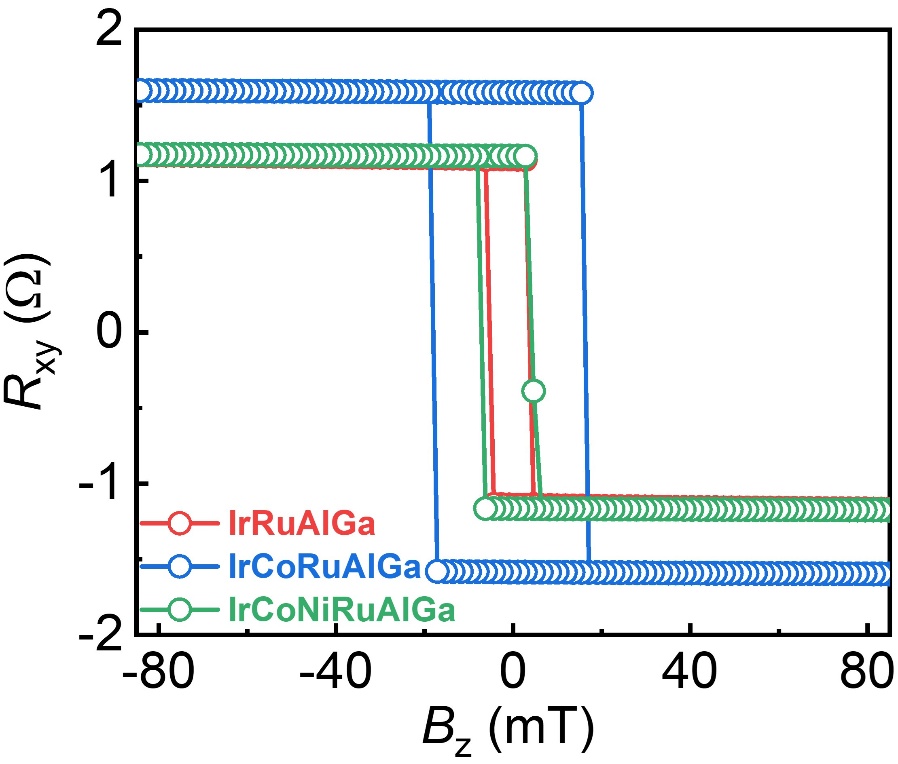


**Figure S13.** Anomalous Hall resistance versus out-of-plane magnetic field of Hall bar devices made from films with structure 50 HEA | 5 Co **|** 7 Ni **|** 4 Co (all units are in Ångstrom), for different HEAs.

**
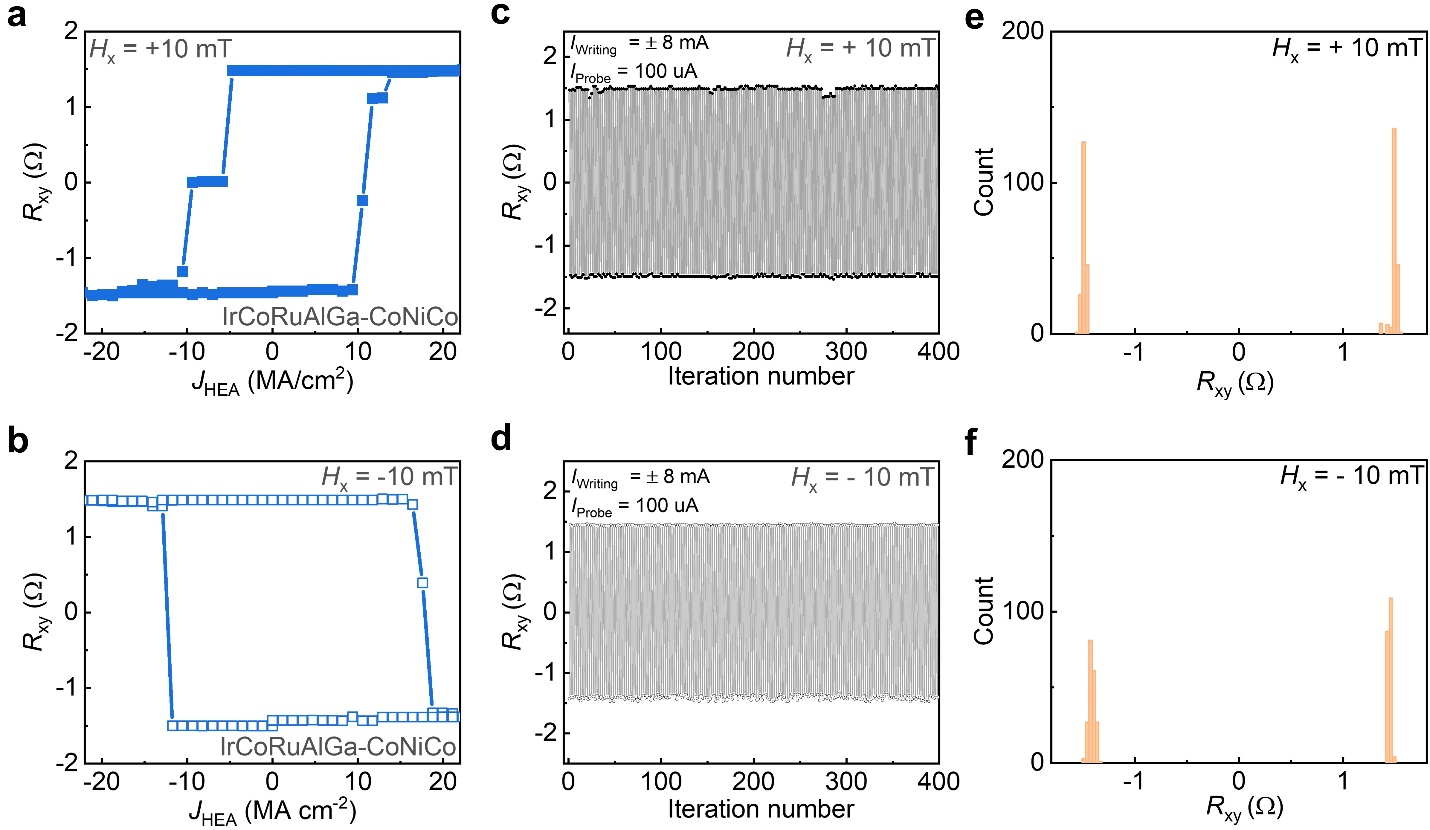
**

**Figure S14. a**,**b**) Spin-orbit torque induced magnetization switching of a Hall bar device made from a film with structure 50 IrCoRuAlGa **|** 5 Co **|** 7 Ni **|** 4 Co (all units are in Ångstrom), with the assistance of an external in-plane magnetic field of **±**10 mT (current pulse length is 1 s). **c,d)** *R*_xy_ switched by a series of positive and negative current pulses as a function of iteration number with assistance of an external in-plane magnetic field of **±**10 mT. **e**,**f)** Deterministic switching of *R*_xy_ with assistance of external in-plane magnetic field of **±**10 mT.

**
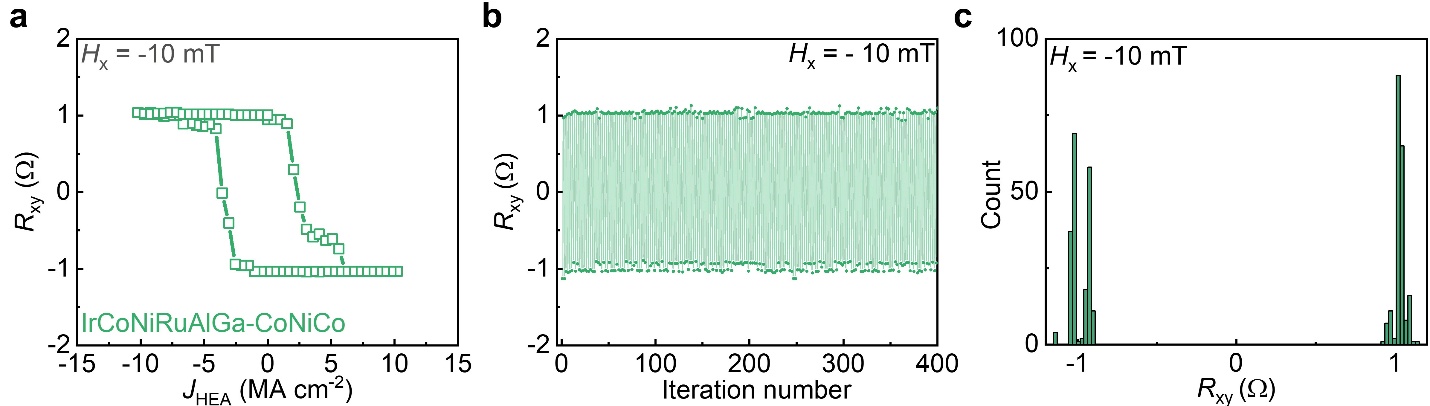
**

**Figure S15. a**) Spin-orbit torque induced magnetization switching of a Hall bar device made from a film with structure 50 IrCoNiRuAlGa **|** 5 Co **|** 7 Ni **|** 4 Co (all units are in Ångstrom) with assistance of external in-plane magnetic field of **-**10 mT with a current pulse length of 1s. **b)** *R*_xy_ switched by a series of positive and negative current pulses as a function of iteration number with assistance of an external in-plane magnetic field of **-**10 mT, respectively. **c)** Deterministic switching of *R*_xy_ with assistance of an external in-plane magnetic field of **-**10 mT.

**
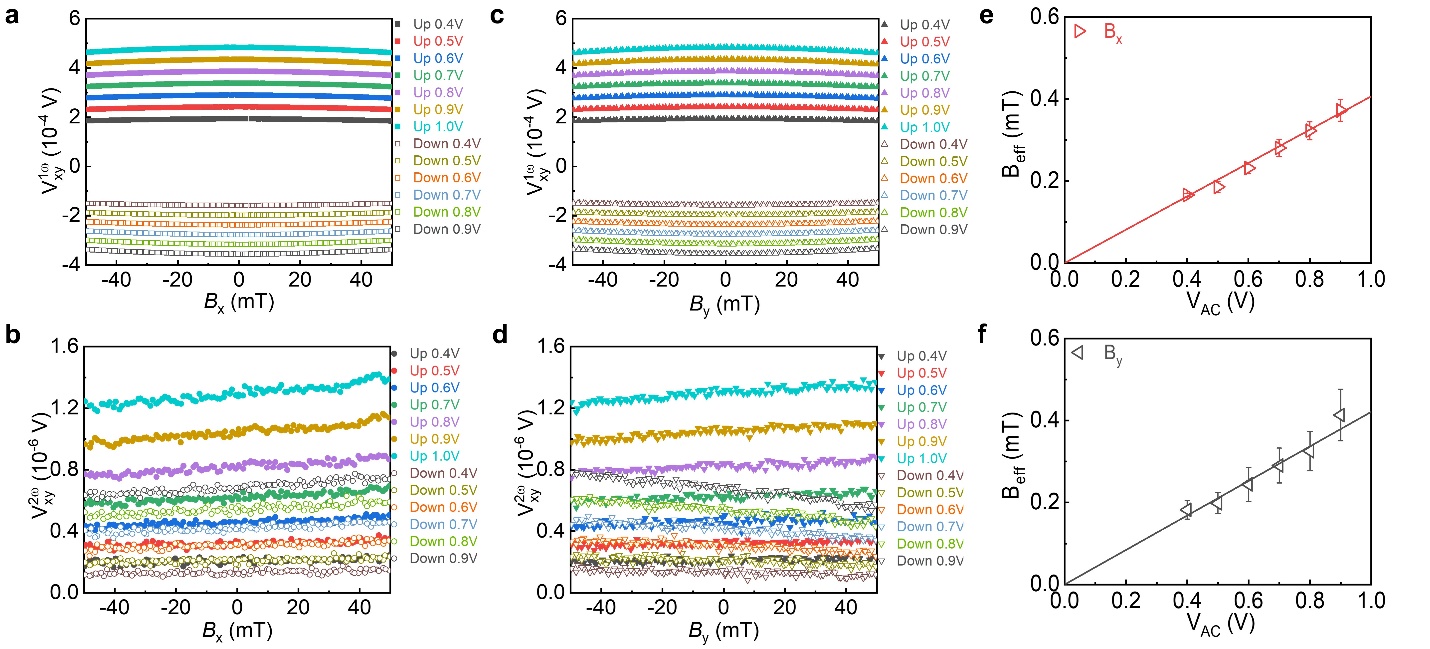
**

**Figure S16. Harmonic Hall measurements for HEA = IrRuAlGa. a)** $V_{\mathrm{xy}}^{1\omega}$ and **b)** $V_{\mathrm{xy}}^{2\omega}$ measured upon applying an alternating voltage (*V*_AC_), with the external magnetic field parallel to the direction of the applied current and **c)** $V_{\mathrm{xy}}^{1\omega}$ and **d)** $V_{\mathrm{xy}}^{2\omega}$ measured with the external magnetic field transverse to the direction of the applied current (Up and Down refer to the magnetization direction of the Co | Ni | Co film being positive or negative along *z*, as shown in Fig. 3a). **e)** B_x_ and **f)** B_y_ effective fields versus *V*_AC_ obtained from the harmonic Hall voltage measurements.

**
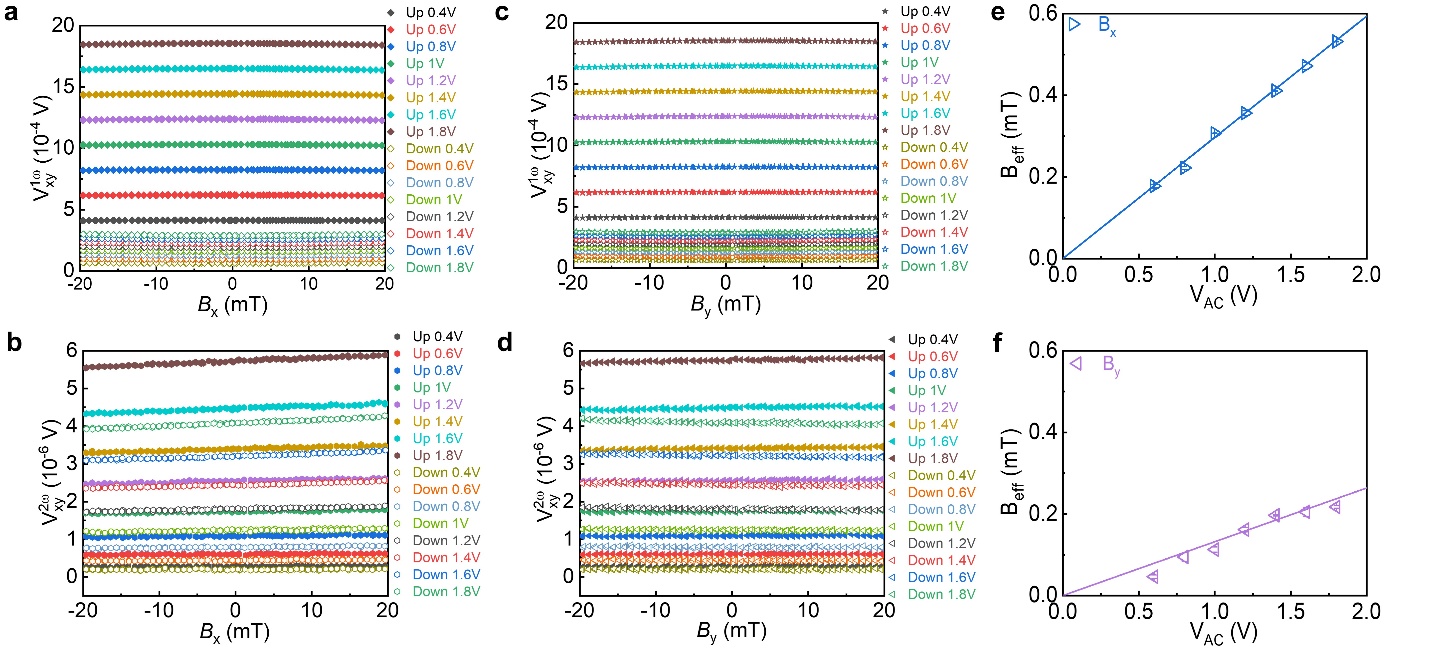
**

**Figure S17. Harmonic Hall measurements for HEA = IrCoRuAlGa. a)** $V_{\mathrm{xy}}^{1\omega}$ and **b)** $V_{\mathrm{xy}}^{2\omega}$ measured upon applying an alternating voltage (*V*_AC_), with the external magnetic field parallel to the direction of the applied current and **c)** $V_{\mathrm{xy}}^{1\omega}$ and **d)** $V_{\mathrm{xy}}^{2\omega}$ measured with the external magnetic field transverse to the direction of the applied current (Up and Down refer to the magnetization direction of the Co | Ni | Co film being positive or negative along *z*, as shown in Fig. 3a). **e)** B_x_ and **f)** B_y_ effective fields versus *V*_AC_ obtained from the harmonic Hall voltage measurements.

**
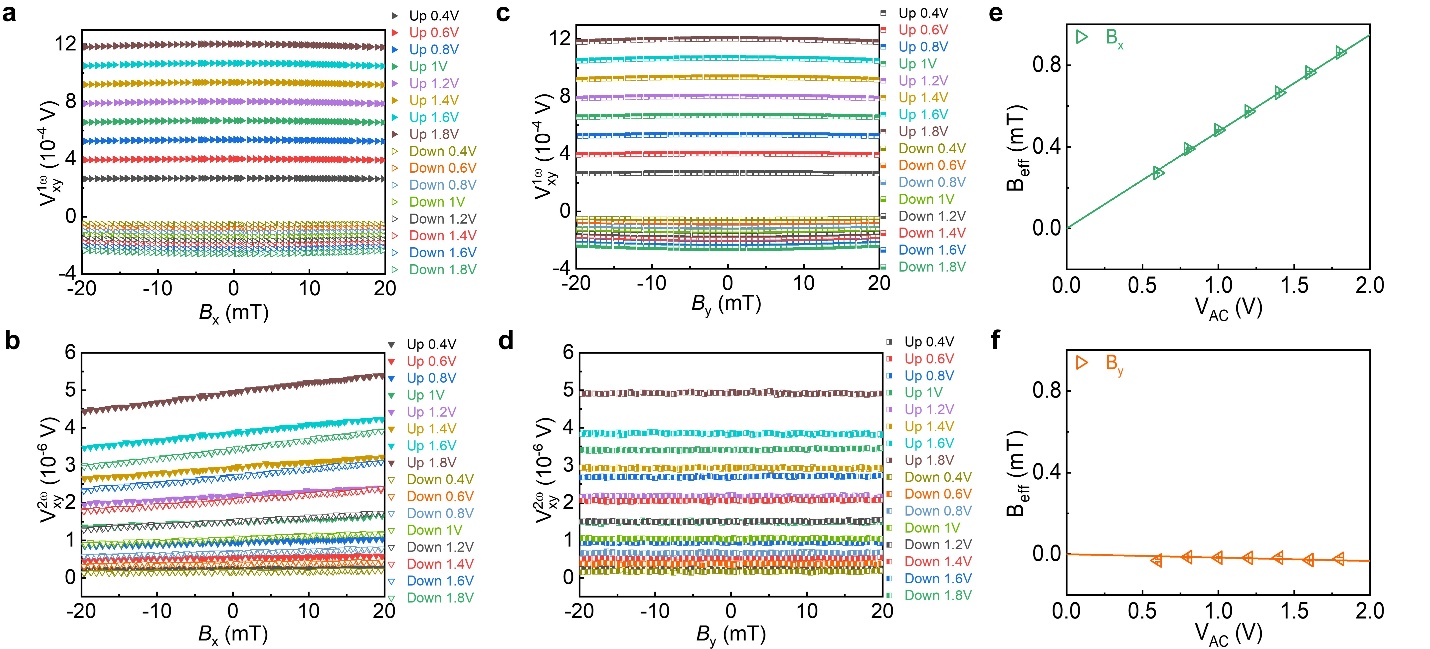
**

**Figure S18. Harmonic Hall measurements for HEA = IrCoNiRuAlGa. a)** $V_{\mathrm{xy}}^{1\omega}$ and **b)** $V_{\mathrm{xy}}^{2\omega}$ measured upon applying an alternating voltage (*V*_AC_), with the external magnetic field parallel to the direction of the applied current and **c)** $V_{\mathrm{xy}}^{1\omega}$ and **d)** $V_{\mathrm{xy}}^{2\omega}$ measured with the external magnetic field transverse to the direction of the applied current (Up and Down refer to the magnetization direction of the Co | Ni | Co film being positive or negative along *z*, as shown in Fig. 3a). **e)** B_x_ and **f)** B_y_ effective fields versus *V*_AC_ obtained from the harmonic Hall voltage measurements.

**
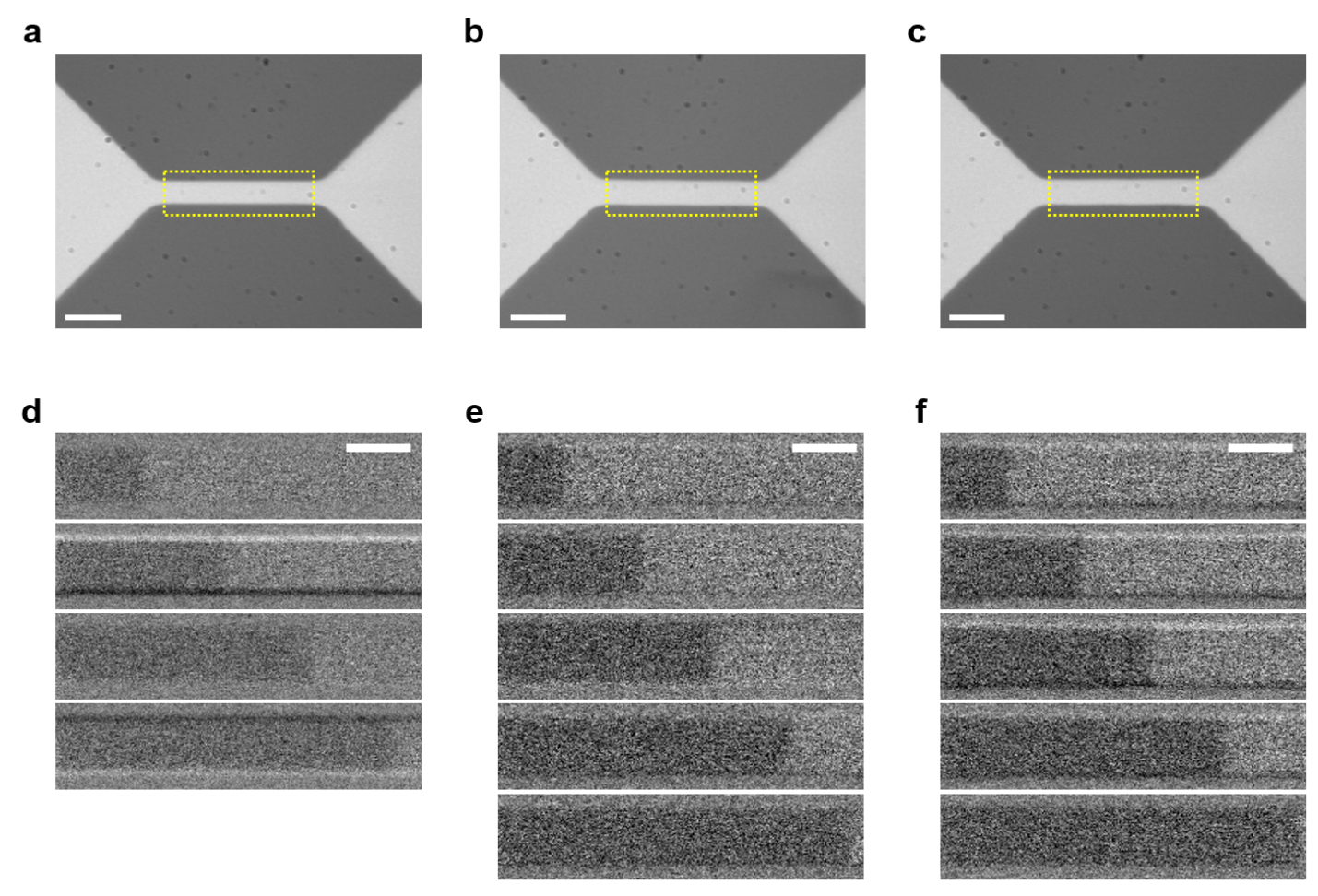
**

**Figure S19. Current-induced domain wall motion in SAFs based on HEA thin films.** Optical microscopy images (**a**-**c**) and differential Kerr microscopy images (d-f) of μm-sized racetracks based on HEA | SAF films, with IrRuAlGa (**a**,**d**), IrCoRuAlGa (**b**,**e**) and IrCoNiRuAlGa (**c**,**f**) as the HEA layer respectively. The Kerr microscopy images of the device region marked with yellow dotted lines in (**a**-**c**), are taken sequentially after injecting 2 current pulses of 80 MA cm^-2^ (**d**) and 6 current pulses of 50 MA cm^-2^ (**e**,**f**) . The scale bars in (**a**-**c**) and (**d**-**e**) correspond to 10 and 5 μm, respectively.

**Notes for second harmonic Hall measurement:**

The second harmonic Hall voltage ($V_{\mathrm{xy}}^{2\omega}$) arises from magnetization oscillations which are induced by the effective fields from SOTs derived from an applied alternating voltage *V* **=** *V*_0_ *sin* *(ωt)*,^[1, 2]^ where *ω* is the angular frequency and *t* is the period. The damping-like (*B*_DL_) and field-like (*B*_FL_) components of the effective magnetic fields induced by SOT can be derived starting from the equation:^[1, 2]^

$B_{x(y)}^{eff}=2\frac{dV_{\mathrm{xy}}^{2\omega}/dH_{x(y)}}{d^{2}V_{\mathrm{xy}}^{1\omega}/dH_{x(y)}^{2}}$ (1)

When the field is swept along the current direction, *B*_x_ can be extracted. *B*_y_ is derived by sweeping the field transverse to the current direction. Considering the ratio of the planar Hall effect resistance (Δ*R*_PHE_) and the anomalous Hall resistance (Δ*R*_AHE_)^[2, 3]^ being:

*ξ=* $\frac{{\Delta R}_{PHE}}{{\Delta R}_{AHE}}$ (2)

One can write:

*B*_DL_= $(\frac{B_{x}^{eff} +2\xi B_{y}^{eff}}{1-4\xi^{2}})$ (3)

*B*_FL_= $(\frac{B_{y}^{eff}+2\xi B_{x}^{eff}}{1-4\xi^{2}})$ (4)

We can quantify the SOT efficiency (*ξ*), namely the damping**-**like (*ξ*_DL_) and field**-**like (*ξ*_FL_) efficiencies as^[4-6]^:

*ξ*_DL(FL)_ = $\frac{2eM_{s}t}{\text{ħ}}$ $\frac{B_{DL(FL)}}{J_{HEA}}$ (5)

where *ħ = h*/2$\pi$, where *h* is Planck’s constant, *e* is the elementary charge, $t$ is the thickness of the magnetic layer, *J*_HEA_ is the current density flowing into the HEA layer, which is calculated based on the parallel resistor model.

*J*_HEA_ = $\frac{I_{HEA}}{S_{HEA}}$= $\frac{I_{HEA}}{W*t_{HEA}}$ = $\frac{I_{HEA}}{W*5 nm}$ (6)

Then according to a simple parallel resistor model and considering that the TaN capping layer is insulating:

*I*_total_ = *I*_HEA_ + *I*_FM_ (7)

Here *I*_HEA_ is the current flowing into HEA layer and *I*_FM_ is the current flowing into the ferromagnetic layer. The sheet resistivities of IrRuAlGa, IrCoRuAlGa and IrCoNi RuAlGa are 216, 242 and 185 μΩ cm, respectively. Furthermore, the resistance of the FM layer can be extracted from the channel resistance of the Hall bar devices used in the harmonic Hall measurement according to their dimensions as shown in Fig. S12. Therefore, we can extract the current density distribution in the different layers using a parallel resistor model:

*I*_HEA_ * *R*_HEA_ = *I*_FM_ * *R*_FM_ (8)

For IrRuAlGa,

*R*_channel_ = 1700 Ω, *L* = 35.2 μm, *w* = 4.2 μm, *t*_HEA_ = 5 nm, *t*_FM_ = 1.6 nm.

3620 *I*_IrRuAlGa_= 3205 *I*_FM_ (9)

*I*_FM_= 1.13 *I*_IrRuAlGa_ (10)

*I*_total_ = 2.13 *I*_IrRuAlGa_ (11)

*I*_IrRuAlGa_ = 0.47 *I*_total_ (12)

*J*_total_= $\frac{I_{toal}}{S_{toal}}$**=**$\frac{I_{toal}}{4.2 \mu m *6.6 nm}$ (13)

*J*_IrRuAlGa_= $\frac{I_{IrRuAlGa}}{S_{IrRuAlGa}}= \frac{0.47 I_{total}}{4.2 \mu m * 5 nm}$ **=** 0.62 *J*_total_ (14)

For IrCoRuAlGa,

*R*_channel_ = 2520 Ω, *L* = 35.2 μm, *w* = 3.1 μm, *t*_HEA_ = 5 nm, *t*_FM_ = 1.6 nm.

5496 *I*_IrCoRuAlGa_= 4653 *I*_FM_ (15)

*I*_FM_= 1.18 *I*_IrCoRuAlGa_ (16)

*I*_total_ = 2.18 *I*_IrCoRuAlGa_ (17)

*I*_IrCoRuAlGa_ = 0.46 *I*_total_ (18)

*J*_total_**=** $\frac{I_{toal}}{S_{toal}}$**=**$\frac{I_{toal}}{3.1 \mu m * 6.6 nm}$ (19)

*J*_IrCoRuAlGa_= $\frac{I_{IrCoRuAlGa}}{S_{IrCoRuAlGa}}= \frac{0.46 I_{total}}{3.1 \mu m *5 nm}$ **=** 0.6 *J*_total_ (20)

For IrCoNiRuAlGa,

*R*_channel_ = 1920 Ω, *L* = 35.3 μm, *w* = 3.5 μm, *t*_HEA_ = 5 nm, *t*_FM_ = 1.6 nm.

3732 *I*_IrCoNiRuAlGa_= 3954 *I*_FM_ (21)

*I*_FM_= 0.94 *I*_IrCoNiRuAlGa_ (22)

*I*_total_ = 1.94 *I*_IrCoNiRuAlGa_ (23)

*I*_IrCoNiRuAlGa_ = 0.52 *I*_total_ (24)

*J*_total_= $\frac{I_{toal}}{S_{toal}}$=$\frac{I_{toal}}{3.5 \mu m *6.6 nm}$ (25)

*J*_IrCoNiRuAlGa_= $\frac{I_{IrCoNiRuAlGa}}{S_{IrCoNiRuAlGa}}= \frac{0.52I_{total}}{3.5 \mu m * 5 nm}$ = 0.69 *J*_total_ (26)

Note that *ξ=* $\frac{{\Delta R}_{PHE}}{{\Delta R}_{AHE}}$ *=* 0.32, 0.19, and 0.29 for IrRuAlGa, IrCoRuAlGa, IrCoNiRuAlGa respectively.

**ST-FMR analysis:**

The RF current applied to the HEA layer generates spin torques that give rise to the precession of the magnetization of the CoFeB layer. The rectified dc voltage drop along the channel, *V*_mix_, arises from the anisotropic magnetoresistance of the CoFeB layer as the magnetization direction is changed via the SOT.

An FMR signal thus results as the magnitude of the in plane magnetic field, *B*_ext_, is varied at a fixed angle.^[7-9]^ The variation of *V*_mix_ with *B*_ext_ can be fitted by the equation:

$V_{mix}=V_{0} [V_{S} \frac{\Delta^{2}}{\Delta^{2}+{(B_{ext}- B_{res})}^{2}}+V_{A} \frac{\Delta(B_{ext}- B_{res})}{\Delta^{2}+\left( B_{ext}- B_{res} \right)^{2}}$] (1)

where *V*_0_ is a pre-factor, *V_S_* and *V*_A_ are the symmetric and antisymmetric magnitudes of the Lorentzian response, *B*_res_ is the resonance field and$\Delta$ is the linewidth.^[10]^

The amplitudes of the symmetric part (*V*s) and antisymmetric part (*V*_A_) are in proportion to current-induced in-plane torque (*τ*_||_) and out-of-plane torque (*τ*_⊥_),^[9, 11]^ respectively, and following the derivation provided in previous studies^[11-12]^, we can estimate the effective charge-to-spin conversion efficiency for y-spin polarization (*θ*_sh,y_) using the equation:

$\theta_{sh,y}=\frac{J_{\sigma,y}}{J_{c}}=\frac{V_{s}\left( +B \right)- V_{s}\left( -B \right)}{V_{A}\left( +B \right)+ V_{A}\left( -B \right)} \frac{e\mu_{0}M_{s}td}{\hbar} \sqrt{1+\frac{\mu_{0}M_{eff}}{B_{res}}}$ (2)

where *t* and *d* are the thicknesses of the CoFeB and HEA layers, respectively, *M*_s_ is the saturation magnetization of the CoFeB layer with 1500 kA m^-1^, and *μ*_0_*M*_eff_ is the effective demagnetization of the sample shown in Fig. S9.

Note that refs. 11 and 12 use different coordinate systems to describe the ST-FMR experiment. In ref. 12, polar coordinates are used to describe the magnetic field B (always positive, rotating by 360°), and the resulting amplitudes of both *V*_A_ and *V*_S_ reverse sign when rotating B by 180°. Conversely, in ref. 11, the magnetic field B is defined with positive and negative values, when rotated by 180°, and therefore only the amplitude of *V*_S_ reverses sign when B is reversed in direction. Here, for the estimation of the effective charge-to-spin conversion efficiency for y-spin polarization (*θ*_sh,y_), we use the coordinate system as given in Ref. 11 (see fig. 2a and S20-21). For the analysis of the angular variation of the ST-FMR response (Fig. 2e-f, S22-24), we use the coordinate system defined in Ref. 12.

As shown in Fig. S20a and b, we set *φ* = 45^o^ between the *I*_RF_ direction and the magnetic field direction, and we extract the *V*_mix_ response as a function of positive (*+B*) and negative (*-B*) magnetic field. $V_{s}\left( +B \right)$ and $V_{s}\left( -B \right)$correspond to the *V*s parameters at positive and negative field *B*, respectively. $V_{A}\left( +B \right)$ and $V_{A}\left( -B \right))$correspond to the *V*_A_ parameters at positive and negative field *B*, respectively.


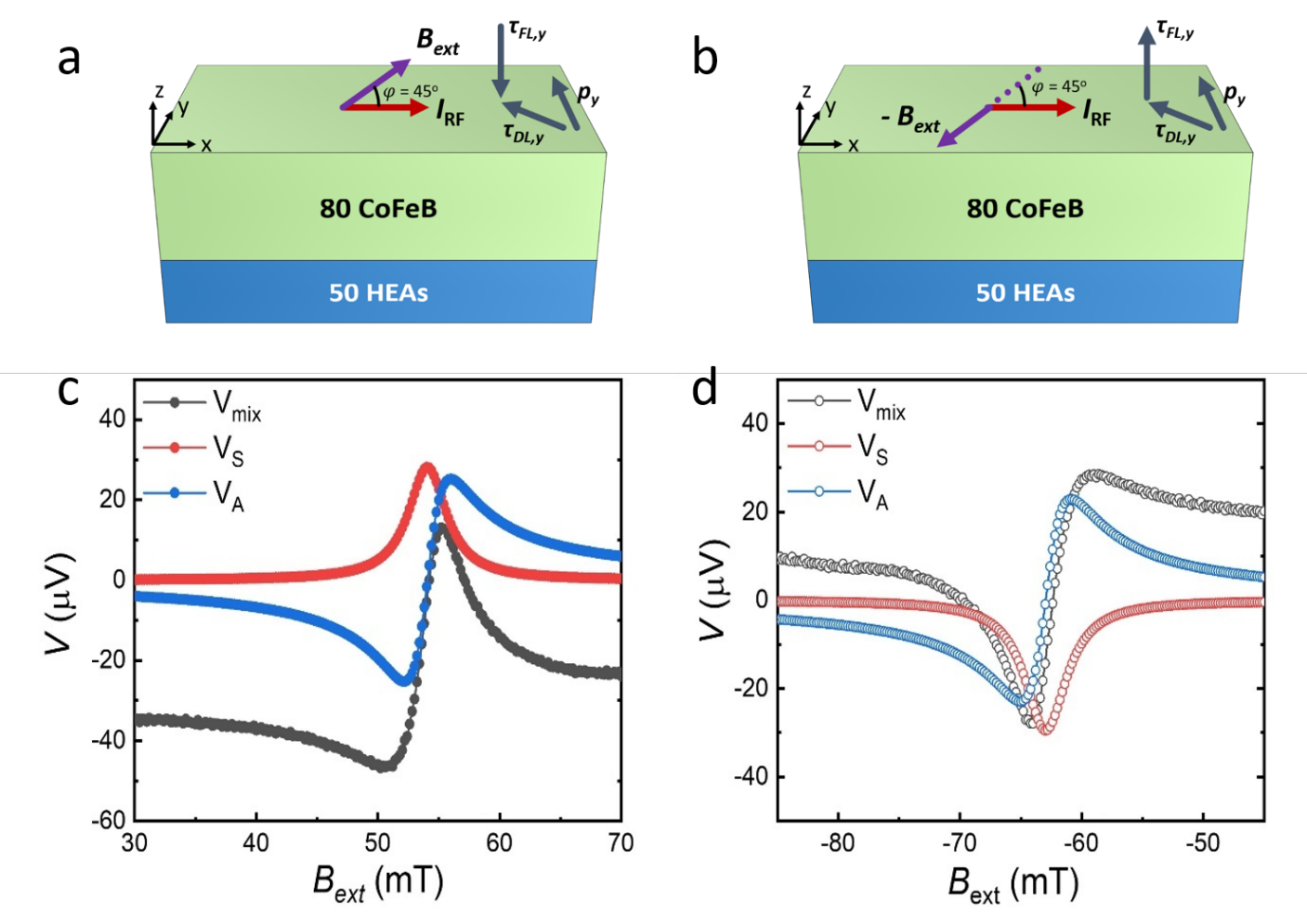


**Figure S20.** **a**,**c**) Schematic of spin-torque ferromagnetic resonance measurements in bilayer thin films of 5 nm HEA **|** 8 nm Co_20_Fe_60_B_20_ stacks. **b**,**d**) ST-FMR spectra measured at 9 GHz for the 5 nm IrCoNiRuAlGa **|** 8 Co_20_Fe_60_B_20_ bilayer thin film with fitting of *V*_S_ and *V*_A_ along with fits based on Eqn. 1 with positive and negative magnetic fields.


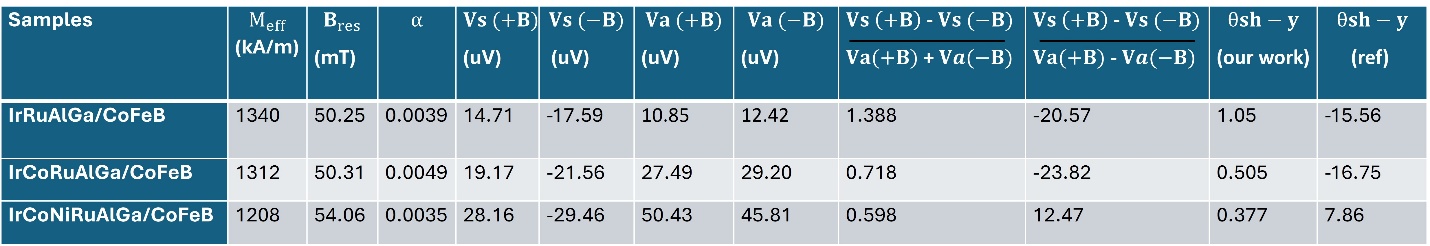


**Figure S21.** Fitting parameters including *V*_S_, *V*_A_, resonant field (*B*_res_), effective demagnetization field (*M*_eff_) and Gilbert damping (α) constant for 5 nm HEA **|** 8 nm Co_20_Fe_60_B_20_ bilayer thin films measured at 9 GHz.

Conversely, when we perform angular dependent ST-FMR measurements, the magnetic field direction is fixed as positive, while the sample is rotated in order to change the angle $\varphi$ between the applied current *I*_RF_ and the external magnetic field *B*. The illustration of the configurations is shown in in Fig. S22.


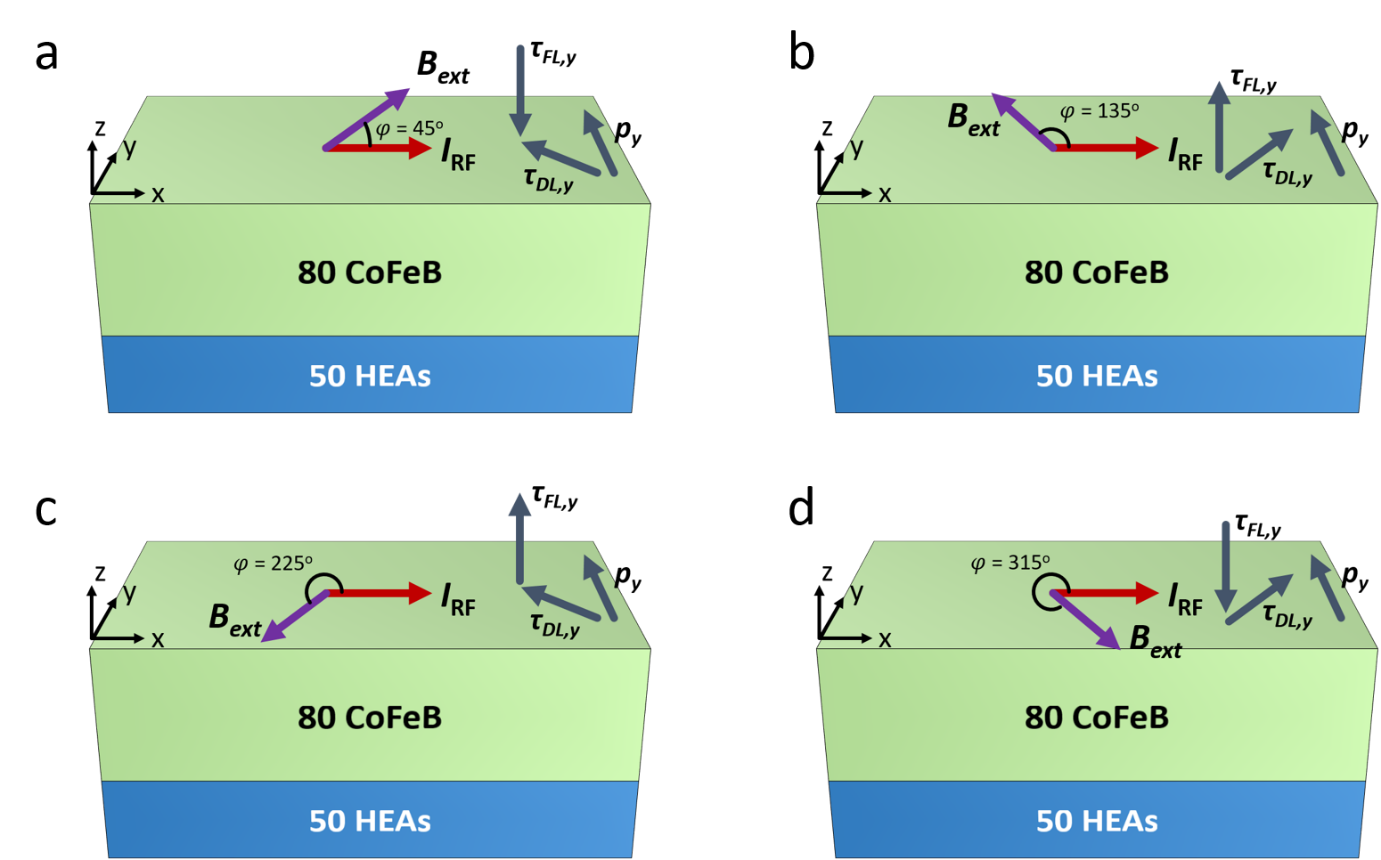


**Figure S22. a-d)** Schematic of angular dependent ST-FMR measurements for for 5 nm HEA **|** 8 nm Co_20_Fe_60_B_20_ bilayer thin films measured at 9 GHz.


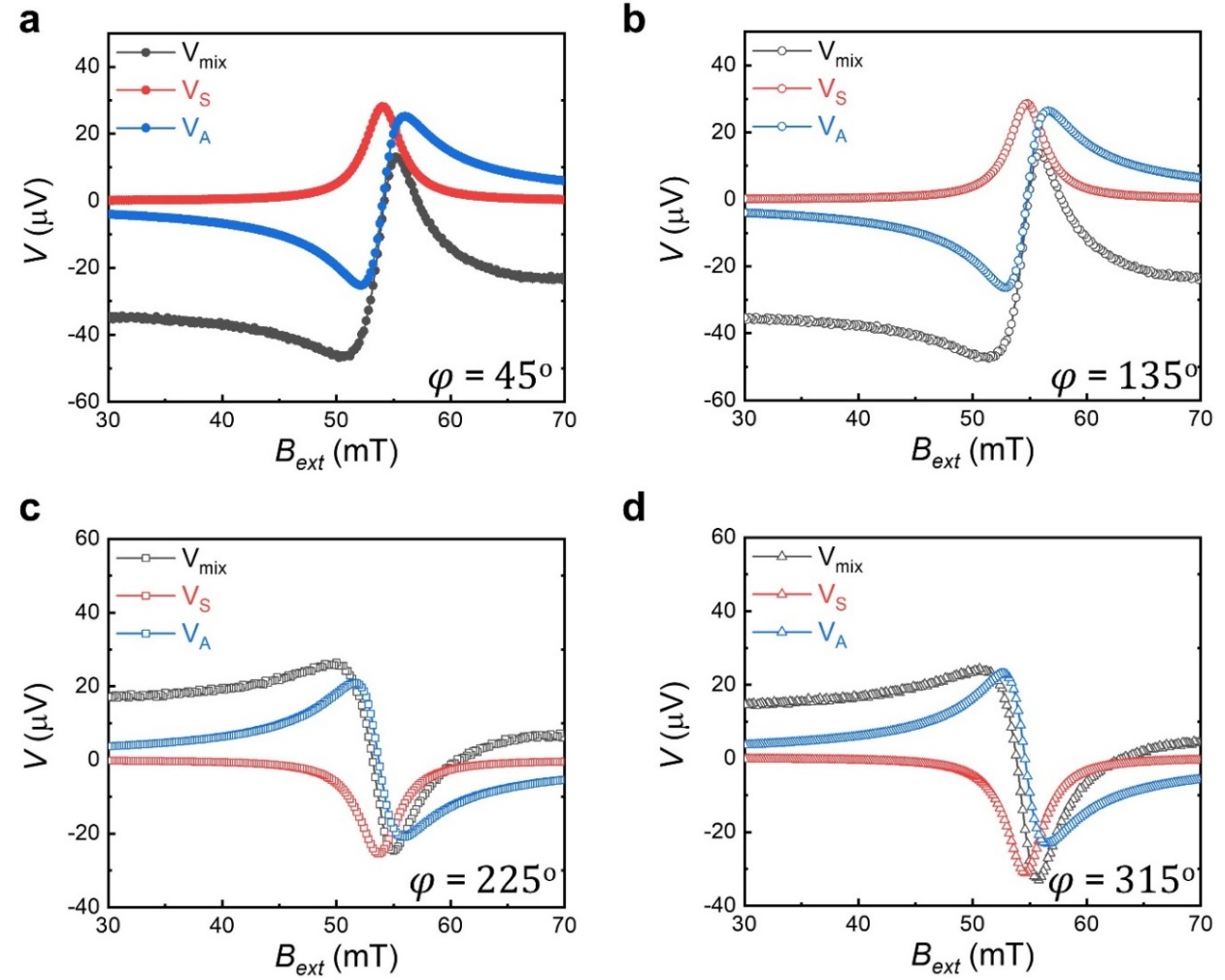


**Figure S23. a**–**d)** ST-FMR d.c. voltages, *V*_mix_, along with fits based on Eq. [1](https://www.nature.com/articles/s41467-023-39884-6#Equ1) for 5 nm IrCoNiRuAlGa **|** 8 nm Co_20_Fe_60_B_20_ bilayer thin films measured at 9 GHz with $\varphi$ =  45°, 135°, 225° and 315°, respectively. The individual *V*_S_ and *V*_A_ contributions are plotted in the figures.


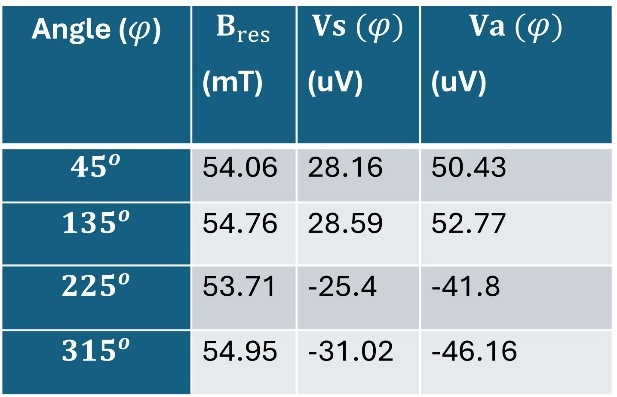


**Figure S24.** Fitting parameters including *V*_S_, *V*_A_, resonant field (*B*_res_), effective demagnetization field for 5 nm IrCoNiRuAlGa | 8 nm Co_20_Fe_60_B_20_ bilayer thin films measured at 9 GHz with $\varphi$ = 45°, 135°, 225° and 315°, respectively.

The high symmetry of our alternating layered tetragonal HEA films do not show strong clear evidence of inversion symmetry breaking and $\tau_{DL,z}$ is negligible from angle dependent ST-FMR measurements, therefore we take $\theta_{sh,y}$ as effective *θ*_sh_ shown in our main manuscript. We also show the calculated results for $\theta_{sh,z}$ below, which, considering the large uncertainty, confirm that $\tau_{DL,z}$ is negligible in these films.


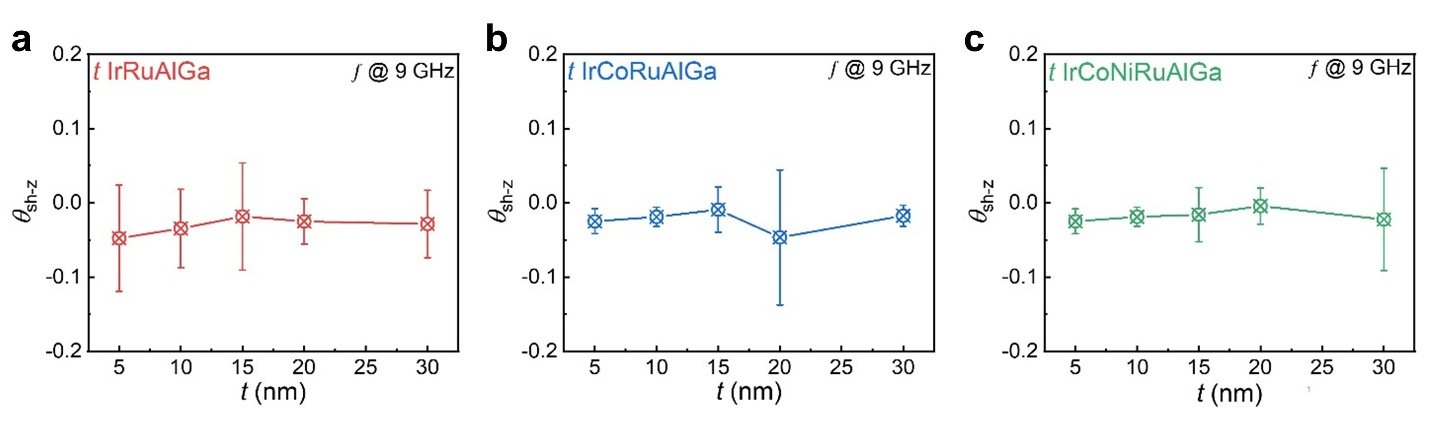


**Figure S25. a-c)** Effective spin Hall angle $\theta_{sh,z}$ obtained at 9 GHz versus HEA thin film thickness.

**References**

[1] J. Kim, J. Sinha, M. Hayashi, M. Yamanouchi, S. Fukami, T. Suzuki, S. Mitani, H. Ohno, *Nat. Mater.* **2013**, *12*, 240.

[2] M. Hayashi, J. Kim, M. Yamanouchi, H. Ohno, *Phys. Rev. B* **2014**, *89*, 144425.

[3] S. Woo, M. Mann, A.J. Tan, L. Caretta, G.S. Beach, *Appl. Phys. Lett.* **2014**, *105*, 212404.

[4] A. Ghosh, K. Garello, C.O. Avci, M. Gabureac, P. Gambardella, *Phys. Rev. Appl.* **2017**, *7* 014004.

[5] M. Dc, D.-F. Shao, V.D.-H. Hou, A. Vailionis, P. Quarterman, A. Habiboglu, M. Venuti, F. Xue, Y.-L. Huang, C.-M. Lee, *Nat. Mater.* **2023**, *22*, 591.

[6] K. Meng, X. Zhang, Y. Chen, X. Deng, T. Zhu, T. Kikkawa, Y. Wu, J. Chen, E. Saitoh, X. Xu, *Adv. Func. Mater.* **2025,** 2426088.

[7] C.-F.P. Luqiao Liu, Y. Li, H. W. Tseng,D. C. Ralph, R. A. Buhrman, *Science* **2012**, *336*, 4.

[8] C.-F. Pai, L. Liu, Y. Li, H.W. Tseng, D.C. Ralph, R.A. Buhrman, *Appl. Phys. Lett.* **2012**, *101*, 122404.

[9] L. Liu, T. Moriyama, D.C. Ralph, R.A. Buhrman, *Phys. Rev. Lett.* **2011**, *106*, 036601.

[10] K.-U. Demasius, T. Phung, W. Zhang, B.P. Hughes, S.-H. Yang, A. Kellock, W. Han, A. Pushp, S.S. Parkin, *Nat. Commun.* **2016**, *7*, 10644.

[11] Y. Liu, G. Shi, D. Kumar, T. Kim, S. Shi, D. Yang, J. Zhang, C. Zhang, F. Wang, S. Yang, Y. Pu, P. Yu, K. Cai, H. Yang, *Nat. Electron.* **2023**, *6*, 732.

[12] D. MacNeill, G. M. Stiehl, M. H. D. Guimaraes, R. A. Buhrman, J. Park, D. C. Ralph, *Nat. Phys.* **2016**, *13*, 300.
